# Preconditioning via Angiotensin Type 2 Receptor Activation Improves Therapeutic Efficacy of Bone Marrow Mononuclear Cells for Cardiac Repair

**DOI:** 10.1371/journal.pone.0082997

**Published:** 2013-12-10

**Authors:** Yinchuan Xu, Xinyang Hu, Lihan Wang, Zhi Jiang, Xianbao Liu, Hong Yu, Zhaocai Zhang, Huiqiang Chen, Han Chen, Gustav Steinhoff, Jun Li, Jian’an Wang

**Affiliations:** 1 Department of Cardiology, the Second Affiliated Hospital, School of Medicine, Zhejiang University, Hangzhou, China; 2 Cardiovascular key lab of Zhejiang Province, the Second Affiliated Hospital, School of Medicine, Zhejiang University, Hangzhou, China; 3 Reference and Translation Center for Cardiac Stem Cell Therapy, University of Rostock, Rostock, Germany; 4 Clinical Stem Cell Research Center and Department of Cardiovascular Surgery, Renji Hospital, Shanghai Jiao Tong University School of Medicine, Shanghai, China; Georgia Regents University, United States of America

## Abstract

**Background:**

The therapeutic efficiency of bone marrow mononuclear cells (BMMNCs) autologous transplantation for myocardial infarction (MI) remains low. Here we developed a novel strategy to improve cardiac repair by preconditioning BMMNCs via angiotensin II type 2 receptor (AT2R) stimulation.

**Methods and Results:**

Acute MI in rats led to a significant increase of AT2R expression in BMMNCs. Preconditioning of BMMNCs via AT2R stimulation directly with an AT2R agonist CGP42112A or indirectly with angiotensin II plus AT1R antagonist valsartan led to ERK activation and increased eNOS expression as well as subsequent nitric oxide generation, ultimately improved cardiomyocyte protection *in* *vitro* as measured by co-culture approach. Intramyocardial transplantation of BMMNCs preconditioned via AT2R stimulation improved survival of transplanted cells in ischemic region of heart tissue and reduced cardiomyocyte apoptosis and inflammation at 3 days after MI. At 4 weeks after transplantation, compared to DMEM and non-preconditioned BMMNCs group, AT2R stimulated BMMNCs group showed enhanced vessel density in peri-infarct region and attenuated infarct size, leading to global heart function improvement.

**Conclusions:**

Preconditioning of BMMNCs via AT2R stimulation exerts protective effect against MI. Stimulation of AT2R in BMMNCs may provide a new strategy to improving therapeutic efficiency of stem cells for post MI cardiac repair.

## Introduction

Myocardial infarction (MI), a main cause of morbidity and mortality, is characterized by myocardium injury, scar formation, and progressive cardiac dysfunction[1]. Stem cells therapy has been identified as a promising approach to MI for their potentiality of differentiation into cardiomyocytes and ability of secretion growth factors and cytokines to nourish myocardium[2]. However, due to low cell engraftment and paracrine activity induced by tough cardiac microenvironment, mechanical injury and maladaptation, stem cells transplantation could only yield marginal benefits (3~4% left ventricular ejection fraction improvement), which severely restrict the clinical potential of this therapeutic approach[3-5]. Previous studies have clearly showed that physical stimulation[6], pharmacological agents treatment[7], genetic manipulation by over-expression of pro-survival related genes[8] could enhance differentiation potential and paracrine activity of stem cells. Therefore, it has been proposed that preconditioning stem cells before transplantation is a good strategy to enhance the therapeutic efficacy of stem cells for post MI cardiac repair[9]. 

Renin-angiotensin (RAS) system was involved in cardiac remodeling after MI[10]. Angiotensin II (AngII), the main effector peptide of RAS, exerts its bioactive effects through angiotensin type 1 receptor (AT1R) and angiotensin type 2 receptor (AT2R). AT2R belongs to the 7 transmembrane G-protein coupled receptor family and exhibits 34% amino acid sequence homology with AT1R. The expression pattern of AT2R is different from AT1R. It is abundant during the fetal development, whereas its expression in adults remains low. By contrast, during tissue injury such as MI and stroke, the expression of AT2R increases dramatically, which is implicated as an important sign in the process of tissue repair[11]. AT2R stimulation through AT1R antagonist indirectly or by AT2R agonist directly could significantly improve cardiac performance after MI, indicating a pivotal role of AT2R in tissue protection[12]. Bone marrow tissue has been widely used as stem cells source for MI therapy. Strawn et al has revealed that all major RAS components including AT2R exist in bone marrow cells, suggesting that AT2R may be a potential target to regulate bone marrow stem cells[13]. Two animal studies has provided evidence that injection of hematopoietic cells or bone marrow stromal cells from AT2R deficient mice led to a significantly neurological deficit in a murine model of brain ischemia compared with cells derived from wide type mice. It appears that AT2R signaling may be required for bone marrow stem cells mediated protection against ischemic brain injury[14,15].

In the present study, we examined the hypothesis whether transplantation of preconditioned bone marrow mononuclear cells (BMMNCs) via AT2R activation could improve overall cardiac performance following MI in rats. Specifically, we addressed the following questions: (1) Does AT2R stimulation indirectly by AngII plus valsartan or directly by CGP42112A enhance cardioprotective effects of BMMNCs *in vitro*? (2) What is the role of AT2R signaling pathway in cardioprotective effects of BMMNCs? (3) Can *in vivo* transplantation of BMMNCs after AT2R stimulation *in vitro* improve heart function? 

## Results

### Increased AT2R Expression in BMMNCs After MI

Because tissue injury could elevate AT2R expression[11], we first compared AT2R mRNA and protein levels in BMMNCs isolated from rats 7 days post-MI with sham operation. Both mRNA and protein abundances of AT2R in BMMNCs were significantly increased as determined by real-time PCR and western blot, respectively (Figure S1 A&B). Therefore, we used rat BMMNCs collected on day 7 post MI as the source of stem cell in this study.

### AT2R Stimulation Improved Cardiomyocyte Protective Effects of BMMNCs *in vitro*

We first co-cultured NRCMs with BMMNCs preconditioned with different dosage of AT2R specific agonist CGP42112A. The percentage apoptotic NRCMs after exposed to hypoxia in serum free medium for 48 hours was then quantified and an optimal dose for suppressing apoptosis was determined. The maximal protective effects can be observed when CGP42112A was delivered at a dose of 10nM (P=0.013 vs BMMNCs group), while 100 nM CGP42112A treatment exhibited a trend of less beneficial effects compared with at a dose of 10 nM, however without reaching a statistical significance (P=0.566 vs 10nM CGP42112A group) (Figure S2 A to F). The protective effect of BMMNCs preconditioned with 10 nM CGP42112A for different time duration was also evaluated to derive an optimal duration. Following different time duration of preconditioning, BMMNCs were co-cultured with NRCMs, and exposed hypoxia and serum free medium, the NRCM survival rate was measured. The protective effect against apoptosis offered by preconditioned BMMNCs was present when BMMNCs have been preconditioned for 1 hour (P=0.044 vs BMMNCs group) and this protective effect was further enhanced when the time duration increased and reached its peak with 2 hours of CGP42112A pre-treatment (P=0.003 vs BMMNCs group) (Figure S3 A to H). Therefore, the dose of 10 nM CGP42112A with a duration of 2 hours was chosen as an ideal BMMNCs preconditioning intervention. 

**Figure 1:**
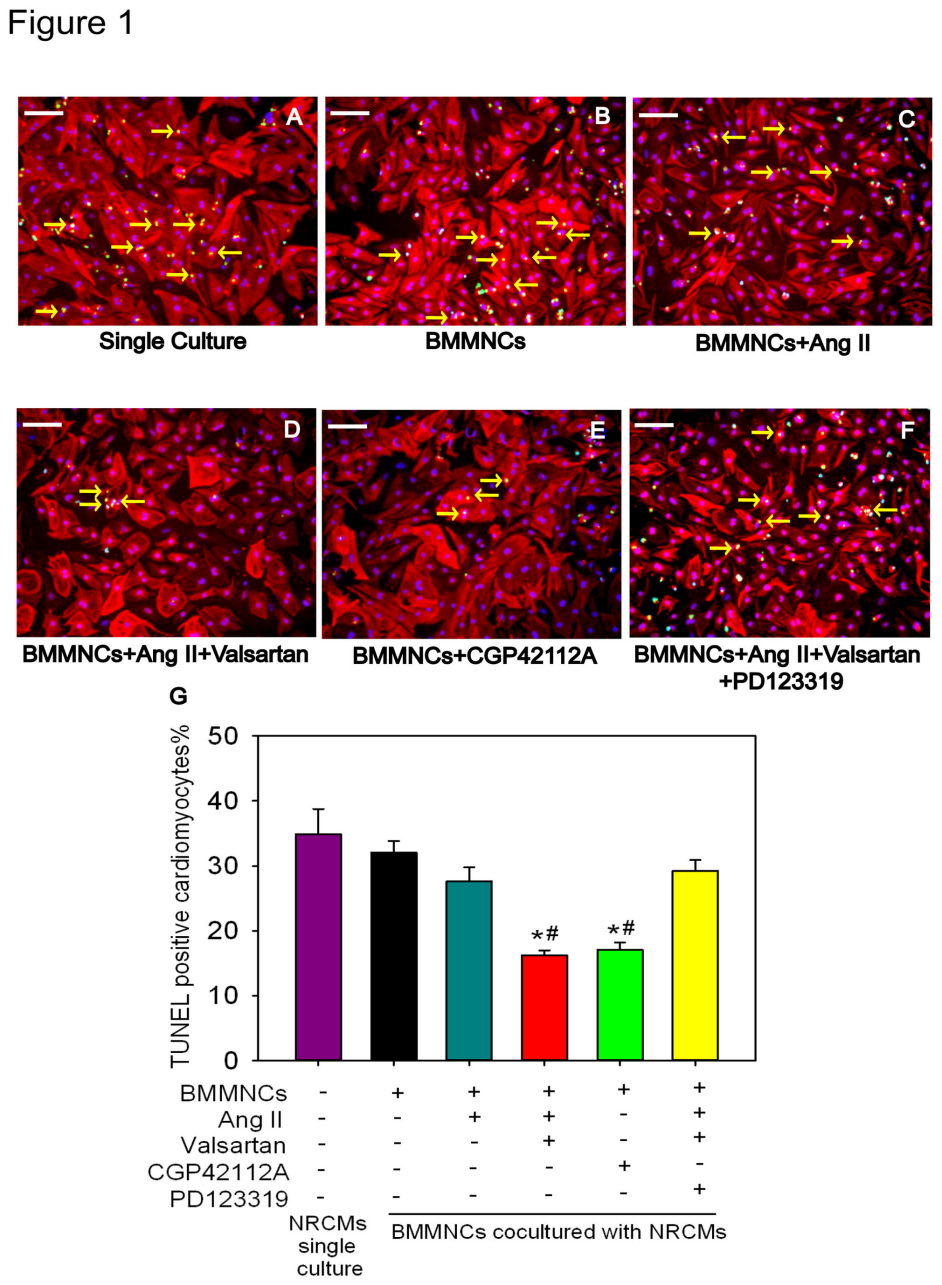
Effect of AT2R Activation on Cardiomyocyte Protection of BMMNCs *in vitro*. (A to F) BMMNCs were initially pre-incubated with DMEM, Ang II, CGP42112A, AngII+Valsartan, or AngII+Valsartan+PD123319 for 2 hours at 37°C, respectively. Then preconditioned BMMNCs were co-cultured with NRCMs under hypoxia in serum free medium for 48 hours. Apoptotic NRCMs were detected using TUNEL assay. Bar=100μm; Green represents TUNEL positive cells; Red represents Troponin T (TnT); Blue represents nuclei; and yellow arrowhead represents apoptotic NRCMs. (G) Quantification of apoptotic NRCMs. n=3 for each group; *P < 0.05 versus NRCMs single culture group and BMMNCs group, ^#^ *P* < 0.05 versus BMMNCs+AngII+Valsartan+PD123319 group.

We next aimed to determine whether differential preconditioning effects would exist between different preconditioning regimen: including 1) partial AT2R stimulation with Ang II; 2) indirect AT2R stimulation by combination of Ang II and AT1R antagonist (Valsartan) in comparison with AT2R agonist. The results showed that when NRCMs co-cultured with BMMNCs that completed different preconditioning regimen had differential resistance to hypoxia and serum deprivation for 48h. NRCMs co-cultured with BMMNCs that did not receive any benefit had a 32.0±1.9% apoptotic cells, which was not significant different from those that were not co-cultured with BMMNCs (apoptotic rate, 34.8±3.9%. P=0.845).BMMNCs pre-treated with AngII alone did not confer any beneficial effects, with apoptotic percentage being at 27.6±2.1% (P>0.05, vs. single culture or BMMNCs group respectively). Interestingly, apoptosis ratio was significantly reduced when co-culture of NRCMs with BMMNCs preconditioned with AngII plus Valsartan, exhibiting similar beneficial effect as those treated with CGP42112A (BMMNCs+AngII+Valsartan group 16.2±0.8% P=0.001 vs BMMNCs group; BMMNCs+CGP42112A group, 17.0±1.2%. P=0.001 vs BMMNCs group). In addition, PD123319, an AT2R blocker, can abolish the protective effects conferred by AT2R stimulation using combination of AngII and AT1R blocker, valsartan, showing an apoptotic ratio of 29.2 ± 1.7% (P<0.05, vs. BMMNCs+AngII+Valsartan group or BMMNCs+CGP42112A group, respectively) which was not different from NRCMs single culture group or BMMNCs group (P>0.05, respectively) (Figure 1 A to G).

### Activation of AT2R/p-ERK/eNOS/NO Signal Pathway in BMMNCs Preconditioned via AT2R Stimulation *in vitro*

**Figure 2:**
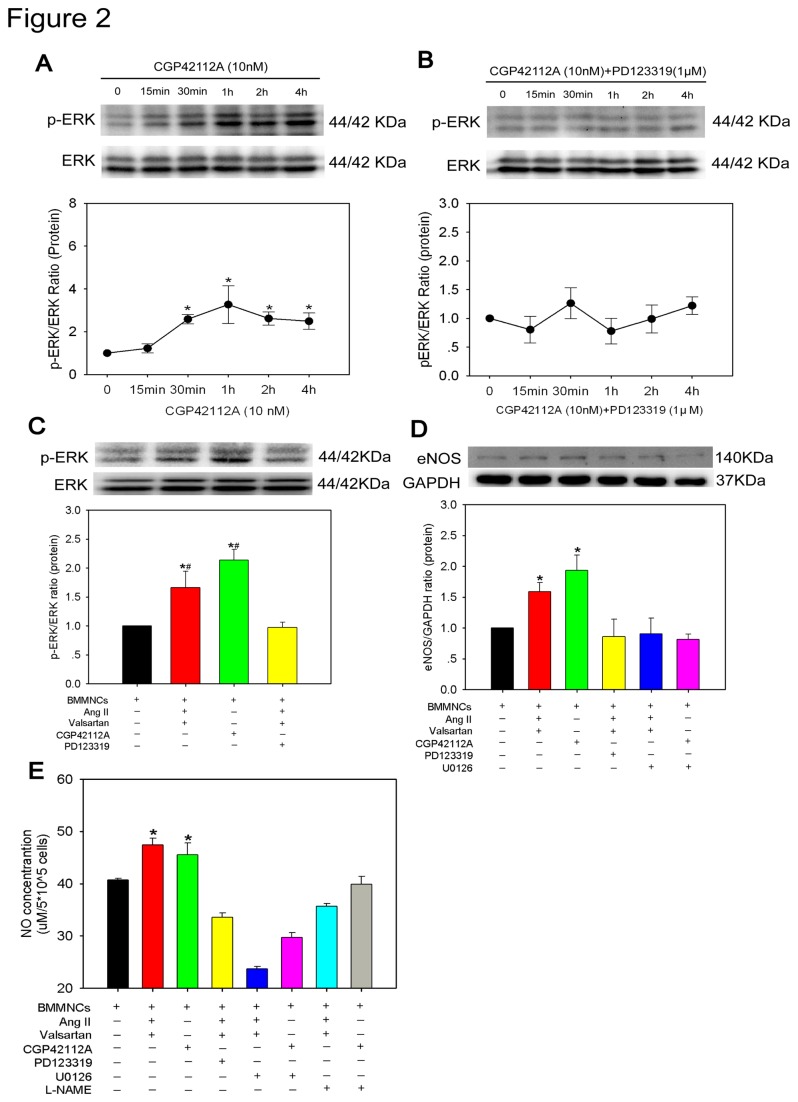
Activation of AT2R/p-ERK/eNOS/NO Pathway in BMMNCs upon AT2R Stimulation. (A) Dynamic change of ERK phosphorylation after 10 nM CGP42112A treatment at different time points. n=3-4 for each group; *P < 0.05 versus baseline (0 min). (B) Dynamic change of ERK phophorylation after 10 nM CGP42112A and 1μM PD123319 co-treatment at different time points. n=5 for each group. (C) Expression of ERK phosphorylation in preconditioned BMMNCs. n=6 for each group; *P < 0.05 versus BMMNCs group, ^#^ *P* < 0.05 versus BMMNCs+AngII+Valsartan+PD123319 group. (D) eNOS expression of preconditioned BMMNCs was assessed by Western blot. n=6 for each group; *P < 0.05 versus all other groups. (E) NO production in cultured medium was determined with a commercial kit. BMMNCs+CGP42112A group n=3; and n=4 for other groups. *P < 0.05 versus all other groups.

**Figure 3:**
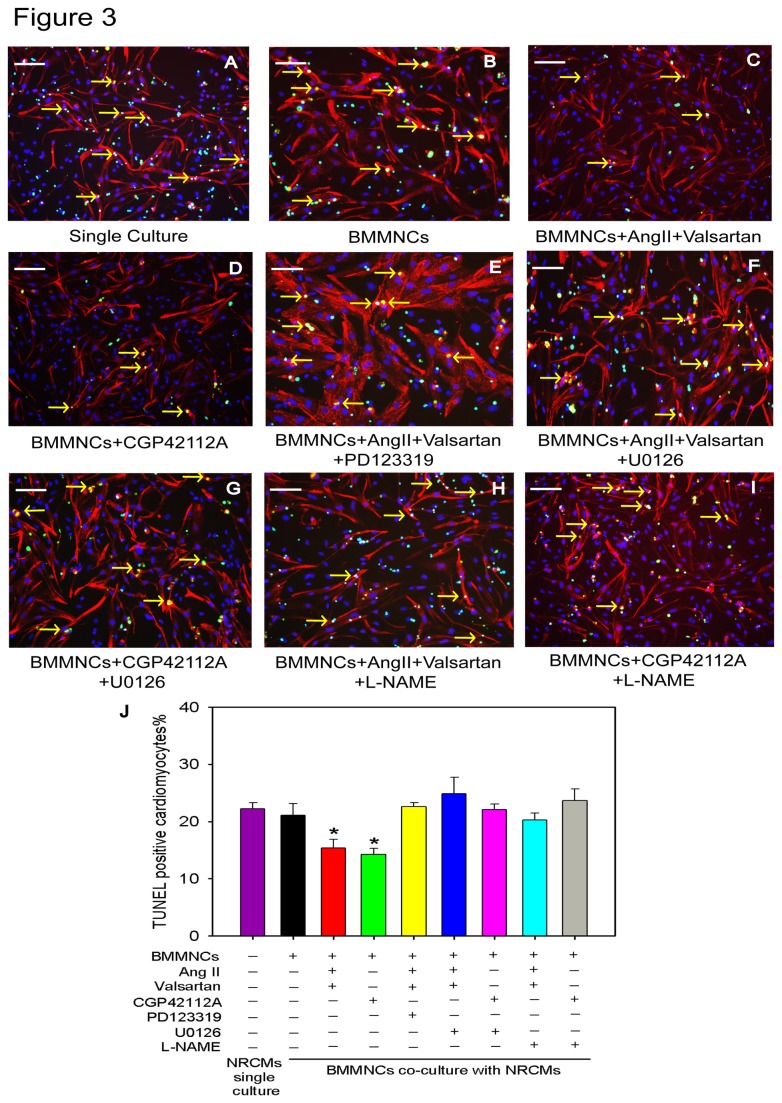
AT2R/p-ERK/eNOS/NO Pathway was Involved in Cardioprotection of BMMNC *in vitro*. (A to I) BMMNCs were initially pre-incubated with DMEM, CGP42112A±U0126, CGP42112A±L-NAME, AngII+Valsartan±U0126, AngII+Valsartan±L-NAME, or AngII+Valsartan+PD123319 for 2 hours at 37 °C, respectively. Then preconditioned BMMNCs were co-cultured with NRCMs under hypoxia in serum free medium for 48 hours. Apoptotic NRCMs were detected using TUNEL assay. Bar=100μm; Green represents TUNEL positive cells; Red represents Troponin T (TnT); Blue represents nuclei; and yellow arrowhead represents apoptotic NRCMs. (J) Quantification of apoptotic NRCMs. Single culture NRCMs group n=6; and other groups n=4 for each group; *P < 0.05 versus all other groups.

It is widely accepted that nitric oxide synthase (eNOS) and nitric oxide(NO) contribute to cadioprotection[16]. Furthermore, it appears that up-regulated eNOS expression and subsequently enhanced NO production are mediated via AT2R activation, involving G alpha i3/Ras/Raf/MAPK (ERK) pathway[17,18]. Therefore, we hypothesized that AT2R/p-ERK/eNOS/NO pathway activation would be account for the cardioprotective effects conferred by preconditioned BMMNCs. To test this hypothesis, we first defined the dynamic change of p-ERK levels after AT2R stimulation. The protein level of p-ERK started to increase at 30 min, reached a peak at 1 hour and maintained at a high level until 4 hours after 10 nM CGP42112A treatment (Figure 2A), thus manifesting a time-course increase in its phosphorylation levels. However, this effect was abolished when AT2R antagonist (PD123319) was added (Figure 2B). Next, we chose 2 hours as preconditioning time duration for each drug to detect the abundance of eNOS and NO expression. BMMNCs showed higher protein levels of p-ERK and eNOS when preconditioned with either CGP42112A or AngII + valsartan, which were ablated when AT2R antagonist, PD123319, was administered (Figure 2C&D). Furthermore, an ERK inhibitor (U0126) attenuated increased eNOS expression, indicating that eNOS was a downstream target of p-ERK (Figure 2D). In addition, we also compared the concentration of nitric oxide (NO) in supernatant of BMMNCs under different stimulations for 24h. Upon activation of AT2R in BMMNCs, NO concentration was significantly increased. However, application of AT2R antagonist (PD123319), ERK inhibitor (U0126), or eNOS inhibitor (L-NAME) significantly attenuated this effect (NO concentration μM/5X10^5^ cells: BMMNCs group 40.7±0.4, BMMNCs+AngII+Valsartan group 47.4±1.3, BMMNCs+CGP42112A group 45.5±2.3, BMMNCs+AngII+Valsartan+PD123319 group 33.6±0.9, BMMNCs+AngII+Valsartan+U0126 group 23.7±0.4, BMMNCs+CGP42112A+U0126 group 29.7±0.9, BMMNCs+AngII+Valsartan+L-NAME group 35.7±0.5, BMMNCs+CGP42112A+L-NAME group 39.9±1.5, P<0.001) (Figure 2E). All these results demonstrated that *in vitro* AT2R stimulation can activate the signal pathway AT2R/p-ERK/eNOS/NO in BMMNCs. To confirm whether each component of AT2R/p-ERK/eNOS/NO pathway was involved in cardioprotection of BMMNC, we performed co-culture assay again. After adding U0126 or L-NAME, the number of apoptotic cardiomyocytes increased (TUNEL^+^ cardiomyocyte percentage: Single culture group 22.2±1.0%, BMMNCs group 21.1±2.0%, BMMNCs+AngII+Valsartan group 15.4±1.5%, BMMNCs+CGP42112A group 14.2±1.1%, BMMNCs+AngII+Valsartan+PD123319 group 22.6 ± 0.7%, BMMNCs+AngII+Valsartan+U0126 group 24.9±2.8%, BMMNCs+CGP42112A+U0126 group 22.1±0.9%, BMMNCs+AngII+Valsartan+L-NAME group 20.2±1.3%, BMMNCs+CGP42112A+L-NAME group 23.7±2.0%, P<0.001) (Figure 3A to J), indicating that the signal pathway AT2R/pERK/eNOS/NO plays a critical role in cardiomyocyte protection of BMMNCs.

### *In vivo* Transplantation of AT2R-stimulated BMMNCs Enhanced Cardiac Repair

**Figure 4:**
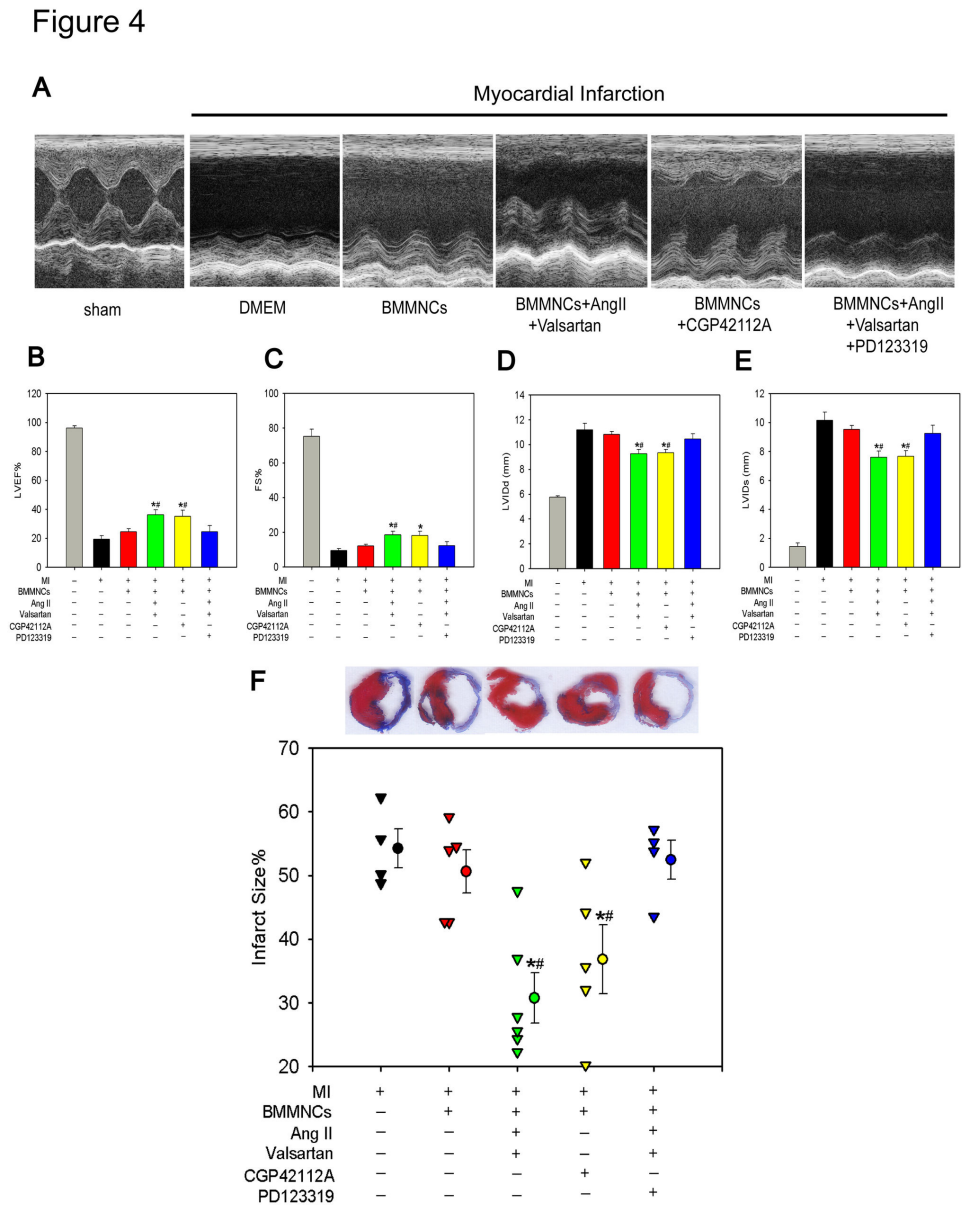
Transplantation of AT2R Stimulated BMMNCs Improved Global Heart Function and Reduced Infarct Size. (A) Representative M-mode images showing cardiac function in each group. (B) Ejection Fraction; (C) Fraction Shortening; (D) LVIDd; and (E) LVIDs. Sham n=5; DMEM group n=8; BMMNCs group n=9; BMMNCs + AngII + Valsartan group n=10; BMMNCs +CGP42112A group n=10; and BMMNCs + AngII + Valsartan + PD123319 group n=9. *P <0.05 versus DMEM group and BMMNCs group, #P < 0.05 versus BMMNCs + AngII + Valsartan +PD123319 group. (F) Intramyocardial transplantation of AT2R stimulated BMMNCs reduced infarct size. DMEM group n=4; BMMNCs group n=5; BMMNCs+AngII+Valsartan group n=6; BMMNCs+CGP42112A group n=5; and BMMNCs+AngII+Valsartan+PD123319 group n=4. *P < 0.05 versus DMEM group and BMMNCs group, ^#^ *P* < 0.05 versus BMMNCs+AngII+Valsartan+PD123319 group.

To determine whether AT2R activation in BMMNCs improves cardiac repair following MI, we performed echocardiography examination on Day 28 after MI. Injection of BMMNCs incubated with CGP42112A or AngII + valsartan resulted in higher ejection fraction (EF) and fraction shortening (FS), and smaller left ventricular internal dimension at end-diastole (LVIDd) and left ventricular internal dimension at end-systole (LVIDs) compared with rats receiving DMEM or non-preconditioned BMMNCs, the beneficial effects were abolished when BMMNCs preconditioned with PD123319 (Figure 4 A to E). Consistent with improved cardiac function, a significant reduction in infarct size was observed in AT2R stimulated BMMNCs that was reversed by AT2R inhibition with PD123319, supporting the notion that AT2R stimulated BMMNCs leads to tissue injury attenuation (Infarct size percentage: DMEM group 54.2±3.1%, BMMNCs group 50.6±3.4%, BMMNCs+AngII+Valsartan group 30.8±4.0%, BMMNCs+CGP42112A group 36.8±5.4%, BMMNCs+AngII+Valsartan+PD123319 group 52.5±3.0%, P=0.001) (Masson trichrome staining shown in Figure 4F).However, there was no difference in survival rate of animals among different groups during the whole observation period (Figure S4).

### AT2R Stimulation Improved Survival of Transplanted BMMNCs In the Region of Ischemic Myocardium *in vivo*

Numerous of evidences indicate that the number of retained exogenous injected cells in the site of infarction is a major factor that affecting the therapeutic efficacy of stem cell transplantation for MI[19]. To determine survival of BMMNCs after transplantation *in vivo* by preconditioning via AT2R activation, we intramyocardial delivered BMMNCs derived from male rats into female rats and took the abundance of sry gene (specific gene for Y chromosome) in heart tissue of female rats as an indirect parameter to reflect the number of lived transplanted cells.

**Figure 5:**
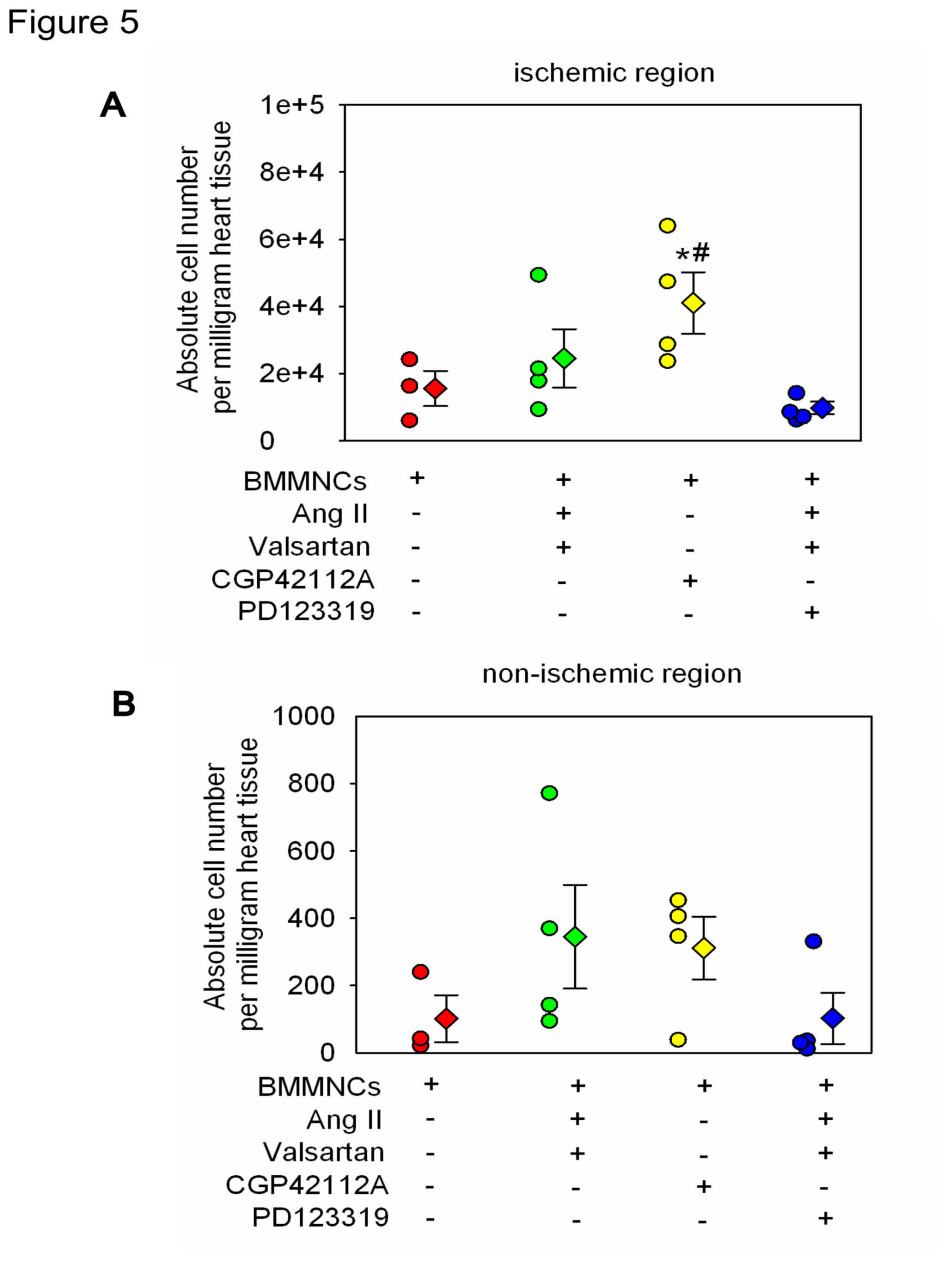
AT2R Stimulation Enhanced Survival of Transplanted BMMNCs in the Region of Ischemic Myocardium *in vivo*. Donor BMMNCs derived from males rats were injected into female rats intramyocardially. 3 days after injection, the abundance of sry gene in ischemic (A) and non-ischemic region (B) of heart tissue was determined by real-time PCR. BMMNCs group n=3; and other groups n=4. *P < 0.05 versus BMMNCs group, and ^#^ *P* < 0.05 versus BMMNCs+AngII+Valsartan+PD123319 group.

As expected, female rats receiving BMMNCs pretreated with AT2R agonist CGP42112A (10nM incubation for 2 hours) exhibited increased copy number of sry gene in the ischemic region of heart tissue, compared with BMMNCs incubated with either DMEM or Ang II+Valsartan+PD123319. Transplantation of AT2R stimulated BMMNCs via AngII plus Valsartan showed the trend of increased cell survival in heart lesion but did not reach the statistical significance (Transplanted cell absolute number per milligram heart tissue: BMMNCs group 15542±5263; BMMNCs+AngII+Valsartan group 24558±8667; BMMNCs+CGP42112A group 40992±9233; BMMNCs+AngII+Valsartan+PD123319 group 9787±1860; P=0.045) (Figure 5A). In addition, AT2R stimulation had no effect on number of lived BMMNCs in the region of non-ischemic heart tissue (Transplanted cell absolute number per milligram heart tissue: BMMNCs group 102±70; BMMNCs+AngII+Valsartan group 345±154; BMMNCs+CGP42112A group 311±93; BMMNCs+AngII+Valsartan+PD123319 group 103±76; P=0.283) (Figure 5B).

### Cardiomyocyte Protection by AT2R Stimulated BMMNCs at early phase of MI *in vivo*

**Figure 6:**
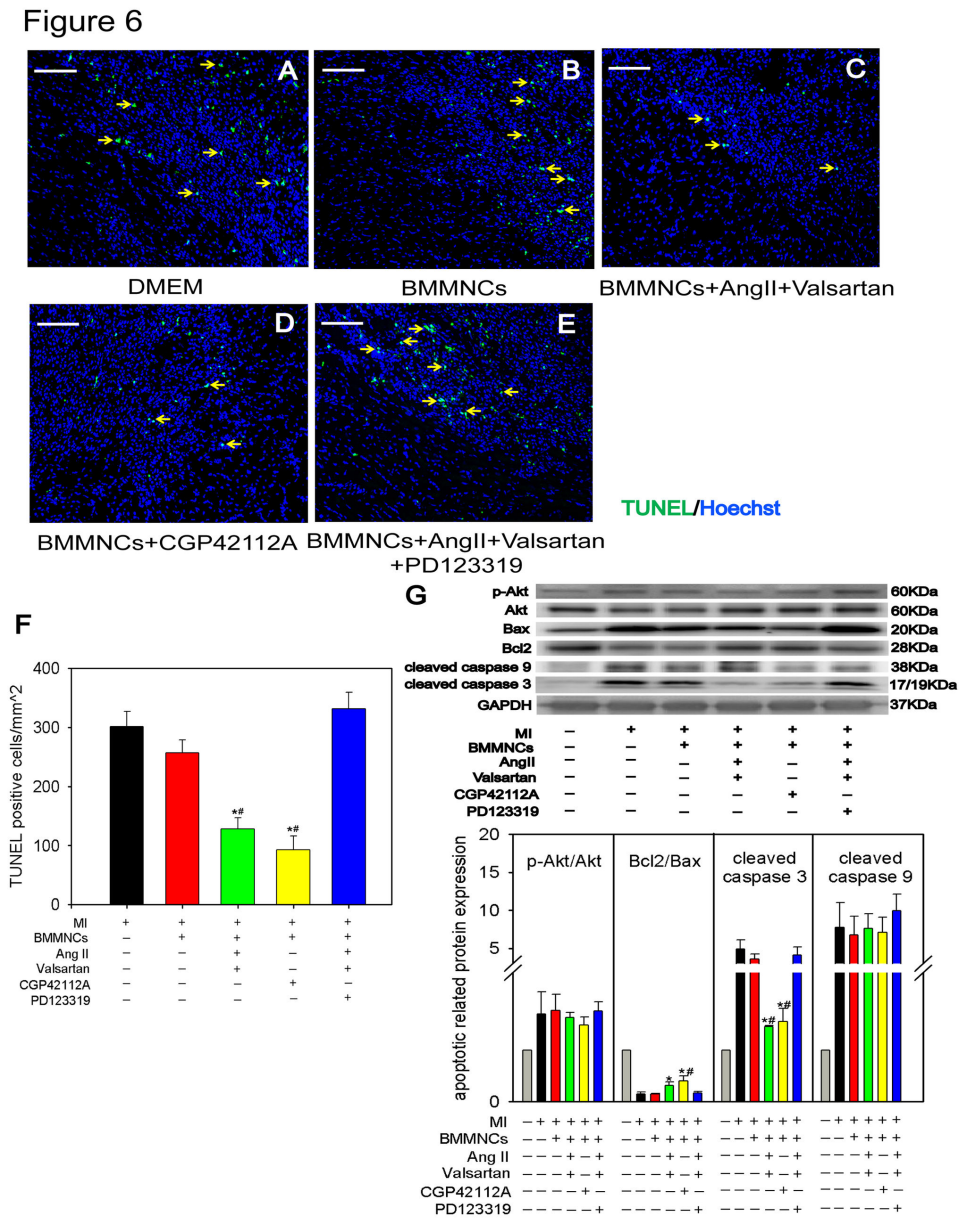
In vivo Effect of AT2R Stimulated BMMNCs on Protection of Adult Cardiac Cells 3 days After Myocardial Infarction. (A to E) TUNEL assay of adult cardiac cells in peri-infarct region of heart tissue. (Bar=100μm; Green represents TUNEL positive cells; Blue represents nuclei; yellow arrowhead represents apoptotic cells). (F) Quantification of TUNEL positive cells. DMEM group n=6; BMMNCs group n=7; BMMNCs+AngII+Valsartan group n=5; BMMNCs+CGP42112A group n=6; and BMMNCs+AngII+Valsartan+PD123319 group n=4. *P < 0.05 versus DMEM group and BMMNCs group, ^#^ *P* < 0.05 versus BMMNCs+AngII+Valsartan+PD123319 group. (G) Western blot analysis of several apoptotic related proteins expression level (Bax, Bcl2, cleaved caspase3, cleaved caspase9, p-Akt, Akt) in border zone respectively. Rat GAPDH was used as control. Quantification of protein level using densitometry analysis (sham n=3; n=3-5 for other each group). *P < 0.05 versus DMEM group and BMMNCs group; ^#^ *P* < 0.05 versus BMMNCs+AngII+Valsartan+PD123319 group.

Once transplanted donor cells aggregated in ischemic heart, the cytokines released by transplanted cells can ameliorate the adverse microenvironment of ischemic myocardium at early stage of MI [20]. To determine the cardiac protective effects of BMMNCs preconditioned with the AT2R agonists *in vivo*, we transplanted BMMNCs intramyocardially into the peri-infarct region. 3 days later, the animals were sacrificed and apoptotic cardiomyocytes were identified by TUNEL staining. Transplantation of BMMNCs preconditioned with AT2R stimulation led to a significant decreased number of TUNEL-positive cells in peri-infarct region, as compared with BMMNCs without any preconditioning, whereas blockade of AT2R with PD123319 abolished the anti-apoptotic effects BMMNCs preconditioned with AngII and valsartan (TUNEL^+^ cells/mm^2^: DMEM group 301±26, BMMNCs group 257±21, BMMNCs+AngII+Valsartan group 128±19, BMMNCs+CGP42112A group 93±24, BMMNCs+AngII+Valsartan+PD123319 group 332±28; P<0.001) (Figure 6 A to F). To determine the potential mechanisms of reduced cellular apoptosis mediated by AT2R stimulated BMMNCs, the apoptotic pathway was analyzed by Western Blot. Cardiac tissues transplanted with AT2R stimulated BMMNCs displayed higher Bcl2/Bax ratio, lower cleaved caspase 3 in the myocardium of border zone compared with those transplanted with DMEM or non-preconditioned BMMNCs. Furthermore, the lower cleaved caspase3 has been reversed after adding AT2R antagonist PD123319. Of note, there were no significant differences in the expression of cleaved caspase 9 and p-Akt (Figure 6G). 

### Intramyocardial Transplantation of AT2R Stimulated BMMNCs Reduced Cardiac Inflammation

**Figure 7:**
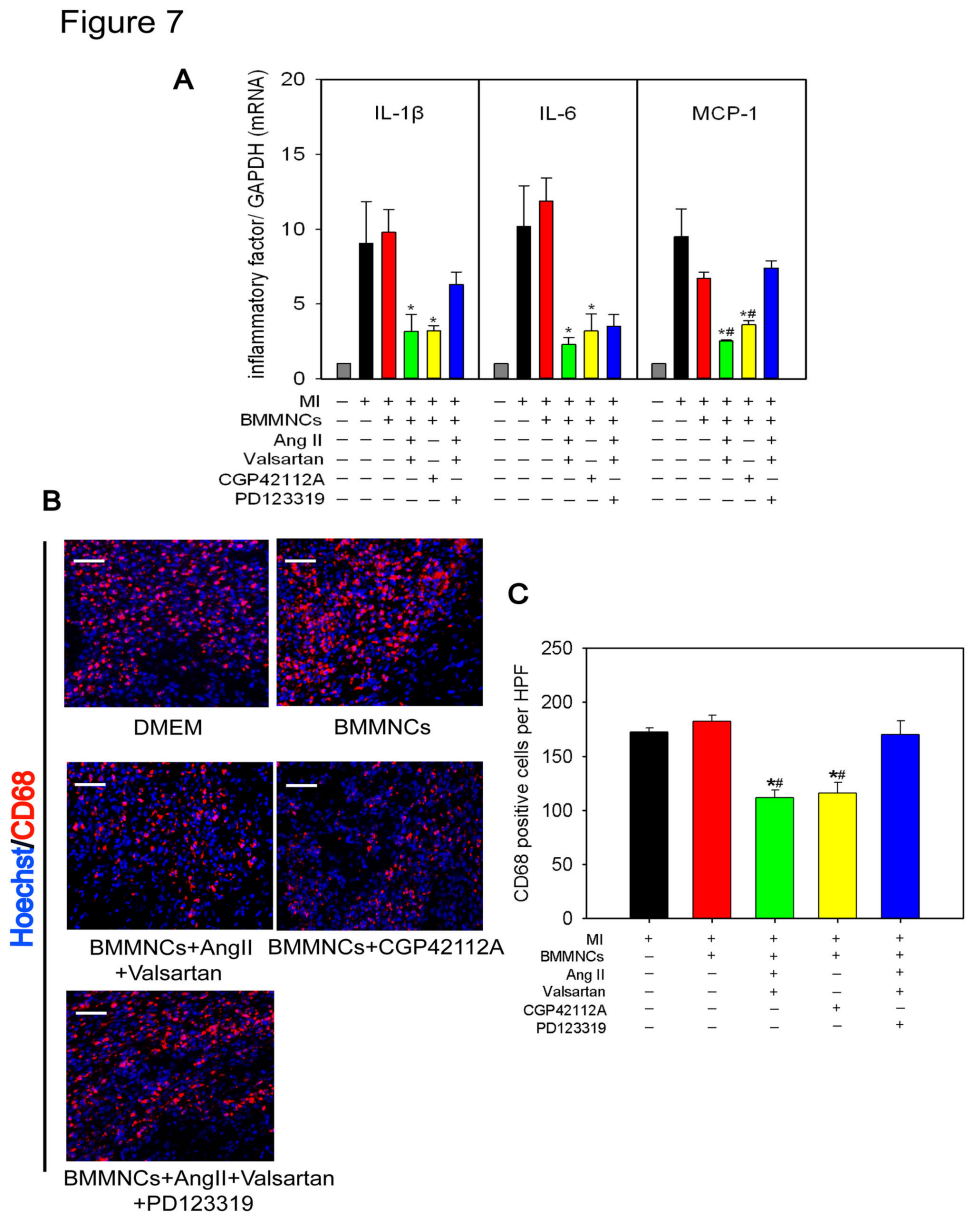
Effect of AT2R Stimulated BMMNCs on Inflammatory Markers of Heart Tissue 3 days After Myocardial Infarction. (A) Real-time PCR of mRNA (IL-1β, IL-6, and MCP-1) expression level in border zone of heart tissues (n=3-4 for each group). *P < 0.01 versus DMEM group and BMMNCs group; ^#^ *P* < 0.01 versus BMMNCs+AngII+Valsartan+PD123319 group. (B &C) Immunofluorescent staining of inflammatory cells (Bar=50μm; Red represents CD68^+^ cells; Blue represents nuclei) in border zone of heart tissues on 3 days post-MI. Bar graph shows quantitative analysis of infiltrating CD68 positive cells at 3 days post-MI (n=4 for each group). *P < 0.001 versus DMEM group and BMMNCs group, ^#^P < 0.001 versus BMMNCs+AngII+Valsartan+PD123319 group.

Inflammation is believed to exert a great influence on prognosis of MI[21]. Furthermore, direct activation of AT2R is associated with attenuation of inflammation in acute phase of MI[12]. Therefore, We also examined whether AT2R stimulated BMMNCs ameliorated cardiac inflammation. AT2R stimulation of BMMNCs markedly reduced inflammatory cytokines IL-1β, IL-6, and MCP-1 in the border zones of myocardium as compared with controls (DMEM alone or non-preconditioned BMMNCs) (Figure 7A). CD68 immunostaining was less abundant in the same area of myocardium injected with BMMNCs preconditioned with AT2R stimulation. Compared with AT2R stimulation, Blockade of AT2R via PD123319 led to higher mRNA abundance of MCP-1 (Figure 7A) and more CD68+ cells (CD68^+^ cells per high power field (HPF): DMEM group 173±4, BMMNCs group 182±6, BMMNCs+AngII+Valsartan group 112±7, BMMNCs+CGP42112A group 116±10, BMMNCs+AngII+Valsartan+PD123319 group 170±13; P<0.001) (Figure 7B&C). 

### Intramyocardial Transplantation of AT2R Stimulated BMMNCs Enhanced Neovascularization

**Figure 8:**
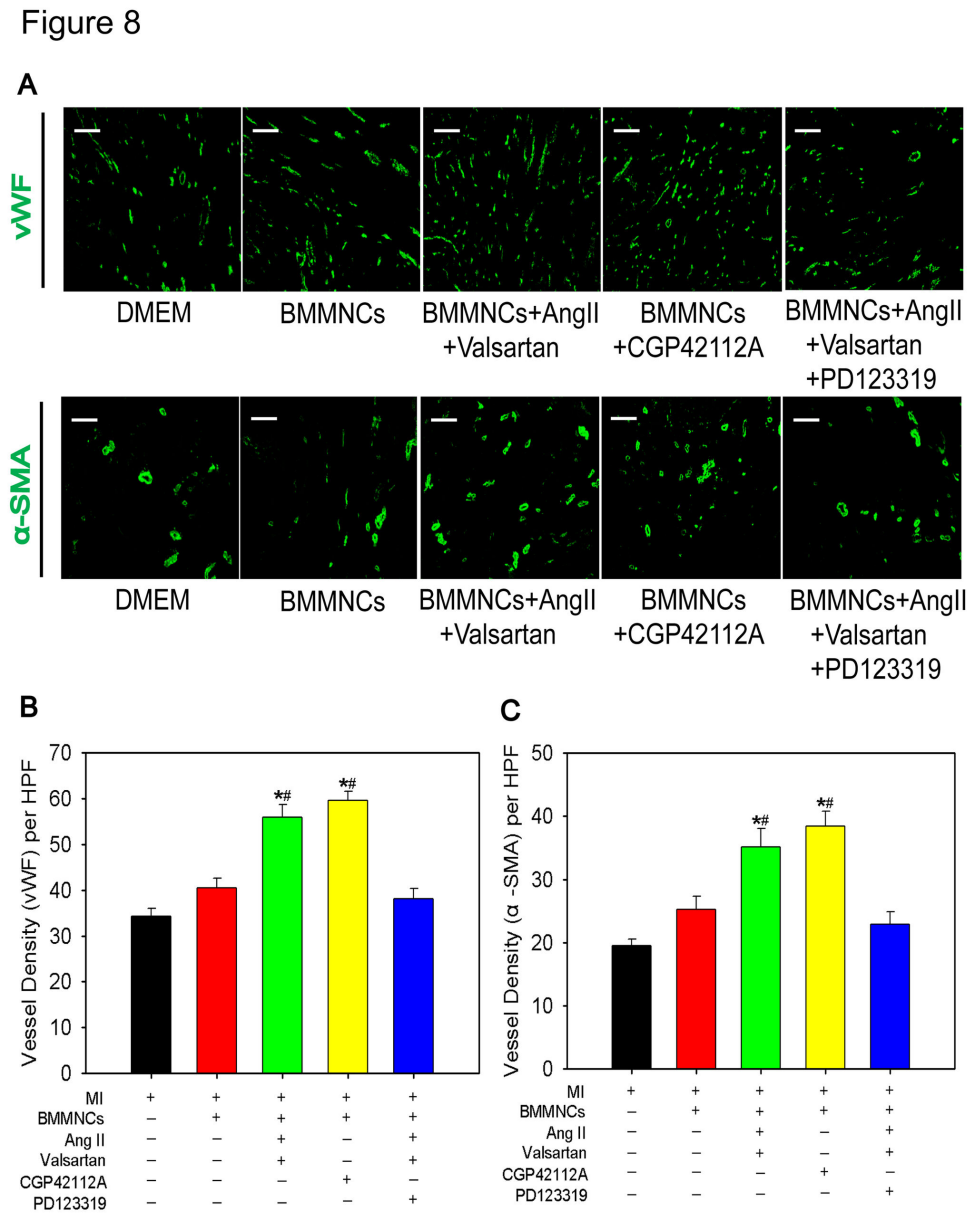
Transplantation of Preconditioned BMMNCs by AT2R Activation Enhanced Angiogenesis in Border Zone on 28 days After MI. (A) Immunofluorescent staining of vWF and alpha-SMA in border zone of heart tissue on 28 days post-MI; (B) Bar graph showed quantitative analysis of vWF positive vessel density. Bar=100μm; DMEM group n=6; BMMNCs group n=4; BMMNCs+AngII+Valsartan group n=5; BMMNCs+CGP42112A group n=4; BMMNCs+AngII+Valsartan+PD123319 group n=5; *P < 0.05 versus DMEM group and BMMNCs group, ^#^ *P* < 0.05 versus BMMNCs+AngII+Valsartan+PD123319 group. (C) Bar graph showed quantitative analysis of alpha-SMA positive vessel density. Bar=100μm; DMEM group n=6; BMMNCs group n=4; BMMNCs+AngII+Valsartan group n=4; BMMNCs+CGP42112A group n=4; BMMNCs+AngII+Valsartan+PD123319 group n=5; *P < 0.05 versus DMEM group and BMMNCs group; ^#^ *P* < 0.05 versus BMMNCs+AngII+Valsartan+PD123319 group.

An important aspect of cardiac repair is neovascularization that is associated with restoration of blood flow and nutrition of myocardium[22]. We evaluated peri-infarct angiogenic and vascularization activity by immunostaining of vWF and alpha-SMA on frozen sections that were harvested from heart tissues 28 days after MI. AT2R stimulation of BMMNCs displayed higher vessel density as compared with DMEM alone or non-preconditioned BMMNCs. In contrast, inhibiting AT2R via PD123319 attenuated the effects induced by AngII + valsartan. (vWF^+^ vessel density per HPF: DMEM group 34±2, BMMNCs group 41±2, BMMNCs+AngII+Valsartan group 56±3, BMMNCs+CGP42112A group 60±2, BMMNCs+AngII+Valsartan+PD123319 group 38±2, P<0.001; alpha-SMA^+^ vessel density per HPF: DMEM group 20±1, BMMNCs group 25±2, BMMNCs+AngII+Valsartan group 35±3, BMMNCs+CGP42112A group 38±2, BMMNCs+AngII+Valsartan+PD123319 group 23±2, P<0.001).These results implicated that transplantation of AT2R stimulated BMMNCs resulted in enhanced angiogenesis during post-MI remodeling process (Figure 8 A to C). 

Similar to the *in vivo* data, *in vitro* angiogenesis assay also showed that HUVEC tube length was significantly increased in the presence of conditioned medium collected from AT2R stimulated BMMNCs, which was abolished when incubated HUVEC with DMEM or conditioned medium from non-preconditioned BMMNCs or BMMNCs treated with AngII+Valsartan+PD123319 at the time point of 2 hours, 4 hours and 6 hours, respectively (Figure S6 A to E and H) .VEGF is the key growth factor in process of angiogenesis, therefore, we measured the concentration of VEGF in supernatant of BMMNCs after AT2R stimulation to determine whether paracrine effect in angiogenesis was caused by AT2R induced VEGF expression. Unexpectedly, neither AT2R stimulation directly (CGP42112A) nor AT2R stimulation indirectly (AngII+Valsartan) altered the concentration of VEGF, indicating that VEGF is not responsible for pro-angiogenesis effect caused by BMMNCs upon AT2R activation. (VEGF concentration (ng/L/5X10^6^ cells): BMMNCs group 779.3±47.4, BMMNCs+AngII+Valsartan group 838.9±27.2, BMMNCs+CGP42112A group 839.4±23.1, BMMNCs+AngII+Valsartan+PD123319 group 855.8±34.9; P=0.445) (Figure S5). Interestingly, when we cultured HUVEC with conditioned medium derived from BMMNCs treated with eNOS inhibitor (L-NAME), we have observed that tube formation was apparently inhibited after adding L-NAME, demonstrating that NO may participate in the process of pro-angiogenesis mediated by AT2R stimulated BMMNCs *in vitro* (Figure S6 F,G and H).

## Discussion

The major finding of the present study is that preconditioning of BMMNCs via AT2R stimulation activates the p-ERK/eNOS/NO pathway, the increase of NO contributes to enhanced cardioprotective effects of BMMNCs *in vitro*. *In vivo* transplantation of AT2R stimulated BMMNCs can enhance survival of transplanted cells in ischemic region, induce cardiac cells apoptosis reduction, inflammation attenuation and angiogenesis increase, leading to improved ventricular remodeling and hence enhanced cardiac performance and geometry.

Autologous cell therapy using bone marrow-derived stem cells has sparked intense hope for cardiac repair. Cumulative animal studies have revealed that BMMNCs transplantation can effectively improve heart function following MI[23,24]. These studies collected BMMNCs from healthy animals, whereas BMMNCs used in our study were from rats with MI. Clinical observations has indicated that cardiovascular disease itself such as MI may significantly impair the functional activity of endogenous stem cells and reduce the efficacy of patient-derived cells for therapeutic purpose[25], which may partly explain why we did not detect any benefit from single BMMNCs transplantation in current study. However, it is interesting that preconditioning of post MI collected BMMNCs via AT2R stimulation can recover their protective effects against MI, suggesting that AT2R signaling might be required for BMMNCs mediated therapy to MI.

AT2R has been identified as a target to tissue repair for its regenerative potential[26]. Preconditioning of human bone marrow mesenchymal stem cells with AT1R antagonists improves efficiency of cardiomyogenic transdifferentiation and heart function, which may be attributable to both direct AT1R inhibition and indirect AT2R activation[27]. We have previously shown that AT2R is induced in post-infarct cardiac c-kit+ progenitor cells and mediates cardiac repair, providing evidence that AT2R contributes to cardiac regenerative process[28]. However, our *in vitro* study showed that co-cultured with AT2R stimulated BMMNCs can protect NRCMs from apoptosis induced by hypoxia and serum free stress. Such effects can be abolished when AT2R antagonist was added, suggesting that AT2R signaling could also promote the paracrine action of BMMNCs which support the survival of NRCMs. Several studies has clearly shown that AT2R stimulation can induce pulmonary artery relaxation through enhanced eNOS expression and NO releasing[17,18]. Pretreatment with eNOS enhancer AVE9488 increases NO generation of bone marrow mononuclear cells and improves their functional activity in a murine model of unilateral hind limb ischemia[29]. Considering the pivotal role of NO in cardiac protection[16], it appears that eNOS and NO may also be involved in protecting NRCMs against hypoxia and serum free injury mediated by AT2R stimulated BMMNCs. In present study, we had shown that AT2R stimulation can enhance the activity of ERK, subsequently increase eNOS expression, thereby promote NO releasing from BMMNCs, leading to protective effects on NRCMs. 

A superior cardiac functional improvement was observed 4 weeks after transplantation of preconditioned BMMNCs compared with DMEM or non-preconditioned BMMNCs. This effect was associated with activation of pro-survival pathways (Bcl2/Bax ratio increase), inhibition of pro-apoptotic pathways (cleaved caspase 3 decrease) in heart tissue. Inflammatory microenvironment is a big challenge for BMMNCs. Cells can be rejected by the inflammatory response, thereby affect their plasticity and long-time survival[30]. Interestingly, we have also found that BMMNCs preconditioned by AT2R exert anti-inflammation effect as shown by decreased cardiac expression of IL-1β, IL-6 and MCP-1, and CD68 positive macrophage infiltration. This is consistent with a previous report from Elena Kaschina et al , that showed down-regulation of IL-1, IL-6 and MCP-1 played a critical role in the protective effect of AT2R agonist on cardiomyocyte after MI[12]. 

In the present study, we have observed that injection of preconditioning BMMNCs via AT2R activation promote neovascularization in peri-infarct zone of heart tissue. In addition, we also showed that tube formation was enhanced when cultured HUVEC with conditioned medium from AT2R stimulated BMMNCs which was contradict to the finding of Ralf Benndorf’s group who reported that AT2R stimulation by CGP42112A can inhibit tube formation of human endothelial cells *in vitro*[31].However, it must be pointed out that the effect of pro-angiogenesis is derived from paracrine factors released by AT2R stimulated BMMNCs, not the direct effect of AT2R activation. Interestingly, we have found that the amount of NO, not VEGF secreted by BMMNCs was increased upon AT2R activation. Furthermore, enhanced angiogenesis induced by BMMNCs upon AT2R stimulation could be partly blocked by eNOS inhibitor *in vitro*, thus it appears that NO may participate in the process of pro-angiogenesis mediated by AT2R stimulated BMMNCs.

Despite the positive results, our study has several limitations. First, although we have found AT2R-mediated cardiomyocyte protection enhancement by BMMNCs are related to the activation of pERK/eNOS/NO pathway *in vitro*, whether this pathway is also involved in cardiac benefit by AT2R stimulated BMMNCs *in vivo* remains unknown. Second, we have observed AT2R stimulation can promote NO generation in BMMNCs. However, we did not determine the change of other growth factors. Therefore, a detailed analysis of paracrine factors profile in BMMNCs upon AT2R stimulation may be of value. Last, BMMNCs is a mixed cell population, determine which cell population within BMMNCs can be responsible to overall cardiac performance improvement upon AT2R stimulation may be necessary. Interestingly, we have previously identified a special cell population named c-kit^+^AT2R^+^ cells existing in both heart and bone marrow tissue, which increases after induction of myocardial infarction. Cardiac c-kit^+^AT2R^+^ cells have the pontency to differentiate into cardiomyocytes and exerts anti-apoptotic effect on cardiomyocytes. However, whether bone marrow c-kit^+^AT2R^+^ cells have the similar characteristic with cardiac c-kit^+^AT2R^+^ cells still remain unknown[28]. Our future studies will specifically address these issues.

In conclusion, preconditioning of BMMNCs by AT2R stimulation promoted transplanted cells and cardiomyocyte survival, attenuated cardiac inflammation, increased angiogenesis, ultimately improved global heart function and reduced infarct size. Our findings suggest that preconditioning by AT2R stimulation may provide a new strategy to improve stem cell therapeutic effects.

## Materials and Methods

### Ethics Statement

All animal studies were performed with approval of the Institutional Animal Care and Use Committee, Zhejiang University.

### Cell Preparation and Treatment

BMMNCs were isolated from rat bone marrow aspiration on day 7 after MI by density gradient centrifugation. In brief, femurs and tibia were harvested from Sprague Dawley rats. Bone marrow aspirates were collected by repeated washing the bone marrow cavity with DMEM (Gibco), and then loaded on Ficoll solution (Tianjin Haoyang Biological manufacture Co.Ltd). Cells were centrifuged at 1800 rpm for 20 min and then transferred the middle one third of total volume into a tube containing DMEM, and centrifuged at 1000 rpm for 10min. After washing with DMEM, cell pellets were suspended in DMEM and then randomly assigned to experimental groups as follows: BMMNCs group (plain control); BMMNCs+AngII (100 nM) group; BMMNCs+AngII (100 nM)+Valsartan (1 μM) group; BMMNCs+CGP42112A (1nM) group; BMMNCs+CGP42112A (10 nM) group; BMMNCs+CGP42112A (100 nM) group; BMMNCs+AngII (100 nM)+Valsartan (1 μM)+PD123319 (1 μM) group; BMMNCs+AngII (100 nM)+Valsartan (1 μM)+U0126 (10 μM) group; BMMNCs+CGP42112A (10 nM)+U0126 (10 μM) group; BMMNCs+AngII (100 nM)+Valsartan (1 μM)+L-NAME (10 μM) group; BMMNCs+CGP42112A (10 nM)+L-NAME (10 μM) group. The incubation time for all these drugs is 2 hours at 37 °C. To determine the optimal time for cell preconditioning, BMMNCs were treated with 10 nM CGP42112A for 30 minute, and 1, 2, 4, and 6 hours at 37°C, respectively. 

The detailed information of drugs is available in Table S1.

### Co-culture Experiments

Neonatal rat cardiomyocytes (NRCMs) were harvested from hearts of neonatal rats using trypsin (Gibco) and preplating to remove fibroblasts. NRCMs culture was maintained in DMEM with 10% FBS. NRCMs (2 x 10^5^/well) were put in the lower chamber of a transwell plate, while either non-preconditioned BMMNCs or drug preconditioned BMMNCs (2 x 10^5^/well) were seeded on the upper chamber of the plate. This co-culture system was placed in a hypoxia incubator with 0.5% oxygen for 48 hours at 37 °C. Single-cultured NRCMs were served as positive control. 

### Determination of Nitric Oxide (NO) Concentration Released by BMMNCs

An NO detection kit (Beyotime) was applied to quantify NO concentration in supernatant of BMMNCs. Briefly, either non-preconditioned BMMNCs or drug preconditioned BMMNCs (5 x 10^5^/well) were seeded in 96-well plates and incubated for 24 hours at 37 °C. After centrifugation, 50 μl Griess reagent i and 50 μl Griess reagent ii were added to 50 μl of supernatant. Nitrite concentration was detected by spectrophotometry (540 nm). NO concentration was expressed as mean ± SEM (nitrite) in μM/5X10^5^ cells.

### Measurement of Vascular Endothelial Growth Factor (VEGF) Concentration Secreted by BMMNCs

Briefly, either non-preconditioned BMMNCs or drug preconditioned BMMNCs (5 x 10^6^/well) were seeded in 24-well plates and incubated for 24 hours at 37 °C. 24 hours later, the supernatant was collected and concentration of VEGF was measured by Rat VEGF ELISA Kit (R&D) following manufacture’s instruction. VEGF concentration was expressed as mean ± SEM ng/L/5X10^6^ cells.

### Tube Formation Assay

Briefly, either non-preconditioned BMMNCs or drug preconditioned BMMNCs (3X10^6^/well) were seeded in 6-well plates and incubated for 24 hours at 37 °C. After centrifugation, the conditioned medium (CM) was collected and used for further angiogenesis assay. Human umbilical vein endothelial cells (HUVEC) were seeded into matrigel prepared wells at the density of 2X10^4^ cells/well in 96-well plates with BMMNCs conditioned medium or control DMEM containing 2% fetal bovine serum. Images were acquired by phase-contrast microscopy at each time point (2 hours, 4 hours, 6 hours and 8 hours).

### Rat Model of Myocardial Infarction

Adult Sprague-Dawlay rats (180-200 g) obtained from Zhejiang Academy of Medical Sciences underwent permanent ligation of the left anterior descending coronary artery (LAD) as described previously[32]. Briefly, animals were anesthetized with 4% chloral hydrate (1ml/100g; Sinopharm Chemical Reagent Co.Ltd) and ventilated with room air by using a small animal ventilator. Myocardial infarction (MI) was induced by permanent ligation of LAD with a 6-0 silk suture. Successful performance of MI model was verified by observing a pale region below the ligation areas. 

### Cell Transplantation Protocol

Immediately after induction of MI in rats, 3 x 10^6^ BMMNCs suspended in 150 μl medium were directly implanted into the border zone. MI control rats received the same volume of DMEM injection. Experimental groups were divided into 5 groups: (1) DMEM group (no cell control); (2) BMMNCs group (cell alone control); BMMNCs preconditioned with (3) AngII(100nM)+Valsartan(1μM) group; (4) CGP42112A(10nM) group; (5) AngII(100nM)+Valsartan(1μM)+PD123319(1μM) group. The preconditioned time for all drugs was 2 hours at 37 °C. 

All heart tissues were harvested on day 3 or day 28 after cell transplantation.

### Echocardiographic Studies

Left Ventricular (LV) function was assessed by transthoracic echocardiography on 28 days after MI using Vevo2100 imaging system. The heart was imaged in two-D and M-Mode. Recordings were obtained from short axis view and LV internal dimension at end-diastole (LVIDd) and LV internal dimension at end-systole (LVIDs) were measured. LV ejection fraction was calculated as follows: LVEF = [LVDEV-LVSEV]/LVDEV. LV fraction shortening (FS) was calculated as follows: FS = [LVIDd-LVIDs]/LVIDd. All measurements were averaged from 5 cardiac cycles.

### Infarct Size Measurement

Masson Trichrome staining and quantification of infarct size were performed as described previously[33]. Briefly, formalin-fixed frozen myocardial sections were stained with Masson trichrome (MaiXin Biological Co.Ltd). Infarct size was evaluated by dividing the sum of the circumferences of endocardium and epicardium of the infarcted area by the sum of the total circumferences of the endocardium and epicardium.

### Detection of Survival of BMMNCs *in vivo*

To evaluate survival of transplanted cells *in vivo*, 1X10^7^ donor BMMNCs derived from male rats with different drugs pretreatment were injected into female rats intramyocardially. On day 3 after transplantation, hearts from the recipient rats were harvested and isolated for genomic DNA using Universal Genomic DNA Extraction Kit Ver.3.0 (Takara). The survival of BMMNCs was determined by quantitative analysis of sry gene (specific gene for Y chromosome) using real-time PCR as previously described[34].

### TUNEL Assay (Terminal Deoxynucleotidyl Transferase Biotin-dUPT Nick End Labeling)

A DeadEnd^TM^ Fluorometric TUNEL System kit (Promega) was applied to evaluate cell apoptosis and viability. After fixing with 10 % formalin for 10 min and treated with chilled ethanol/acetic acid (2:1) and 0.2 % Triton X-100, heart sections or cells were incubated in an EB buffer. Then the TdT enzyme and nucleotide mix were added for 75 min at 37 °C. The slides or cells were washed with 2X SSC for 15 min followed by 3 washes with PBS.

### Western Blot Analysis

Proteins were extracted from cells or heart tissues. 40μg proteins per sample was electrophoresed on a gel (8% or 10% or 12%) and transferred onto a PVDF membrane, and blocked with 5% milk for 1 hour. The membrane was then incubated at 4 °C overnight with primary antibodies as follows: GAPDH, Bax, Bcl2, p-ERK, ERK, eNOS, AT2R, p-Akt, total Akt, cleaved caspase3, and cleaved caspase9 (The detailed information of antibodies is available in Table S1).Then membranes were incubated with HRP-conjugated secondary antibodies at room temperature for 2 hours and exposed with enhanced chemiluminescence (Millipore).

### Real-time PCR

Total RNA was extracted from cells or heart tissues with Trizol reagent (Invitrogen) and then reverse transcripted into cDNA. SYBR Premix Ex Taq kit (Takara) was used for quantification of real-time PCR. Briefly, PCR amplification was carried out in a volume of 20 μl containing 1 μl template, 0.8 μl primers (10 μM, forward and reverse respectively), 0.4 μl ROX, 10 μl SYBR Premix, and 7.8 μl water. PCR samples were denatured at 94°C for 30 s and 40 cycles were performed at 94 °C for 5 s, and 60 °C for 32 s. The value of the cycle threshold was used for calculations. The sequences of primers were presented in Table S2.

### Immunofluorescent Staining

Cells and frozen heart tissues were fixed with 10% formalin for 10 min, 0.2% Triton-X100 for 15 min, and then blocked with 3% BSA for 1 hour. The sections were incubated with primary antibodies overnight at 4 °C as follows: vWF, TnT, alpha smooth muscle actin, CD68 (The detailed information of antibodies is available in Table S1). After washing with PBS for 3 times, the sections were incubated with secondary fluorescent antibodies for 1 hour at room temperature and then incubated with Hoechst 33258 (Invitrogen) for 20 min, and finally analyzed under a fluorescent microscope. For quantification of vessel density, 5 fields within border zone from each animal were obtained. Vessels were analyzed at low magnification, 200X. Quantification of inflammatory cells infiltration (CD68+ cells) was performed as described previously[35]. 

### Statistical Analysis

All data were presented as mean ± SEM. Differences between 2 groups were analyzed with Student two-tailed t-test. Multiple comparisons were done by one-way ANOVA or Two-Way ANOVA using SPSS 16.0 statistical software. *P* < 0.05 was defined as statistically significant.

## Supporting Information

Figure S1 **Elevated AT2R Expression in BMMNCs 7days After MI.** (A) Real-time PCR for AT2R expression in mononuclear cells that were isolated from rat bone marrow on day 7 after MI. Sham n=6; MI n=6; *P < 0.05 versus sham group. (B) Western blot analysis of AT2R expression level of BMMNCs on day 7 after MI. Sham n=3; MI n=3; *P < 0.05 versus sham group.
(TIF)

Figure S2 **Determination of the Optimal Dosage of CGP42112A for BMMNCs Preconditioning.** (A to E) BMMNCs were initially pre-incubated with DMEM, 1nM CGP42112A, 10nM CGP42112A, 100nM CGP42112A for 2 hours at 37 °C, respectively. Then preconditioned BMMNCs were co-cultured with NRCMs under hypoxia in serum free medium for 48 hours. Apoptotic NRCMs were detected using TUNEL assay. Bar=100μm; Green represents TUNEL positive cells; Red represents Troponin T (TnT); Blue represents nuclei; and yellow arrowhead represents apoptotic NRCMs. (F) Quantification of apoptotic NRCMs. n=3 for each group; *P < 0.05 versus BMMNCs group.
(TIF)

Figure S3 **Determination of the Optimal Time Course of CGP42112A for BMMNCs Preconditioning.** (A to G) BMMNCs were initially pre-incubated with 10 nM CGP42112A at different time points (30 min, 1h, 2h, 4h, 6h), respectively. Then preconditioned BMMNCs were co-cultured with NRCMs under hypoxia in serum free medium for 48 hours. Apoptotic NRCMs were detected using TUNEL assay. Bar=100μm; Green represents TUNEL positive cells; Red represents Troponin T (TnT); Blue represents nuclei; and yellow arrowhead represents apoptotic NRCMs. (F) Quantification of apoptotic NRCMs. n=3 for each group; *P < 0.05 versus BMMNCs group.
(TIF)

Figure S4 **The Animal Survival Ratio Among Each Group During Whole Observation Period.**
(TIF)

Figure S5 **Effect of AT2R Activation on VEGF Secretion by BMMNCs *in vitro*.** BMMNCs were incubated with DMEM, CGP42112A, AngII+Valsartan, or AngII+Valsartan+PD123319 for 2 hours at 37 °C, respectively. Then preconditioned BMMNCs were cultured for 24 hours and medium was collected. Concentration of VEGF in supernatant was measured by ELISA. n=4 for each group.
(TIF)

Figure S6 **Conditioned Medium (CM) Derived from AT2R Stimulated BMMNCs Enhanced Tube Formation *in vitro*.** (A to G) Representative figures showed tube formation of HUVECs at time point of 6 hours. Bar=500μm. (H) Quantitative analysis of tube length at each time point. n=3 for each group. *P < 0.05 denotes (BMMNCs+AngII+Valsartan CM group or BMMNCs+CGP42112A CM group) versus all other groups by Two Way ANOVA.
(TIF)

Table S1 **Materials Used in Study.**
(TIF)

Table S2 **Primers Used in Study.**
(TIF)
