# Supplementary figures and images for: Preconditioning via Angiotensin Type 2 Receptor Activation Improves Therapeutic Efficacy of Bone Marrow Mononuclear Cells for Cardiac Repair
Source: PLoS One. 2013 Dec 10;8(12):e82997. doi: 10.1371/journal.pone.0082997 (PMC3858344; doi:10.1371/journal.pone.0082997)

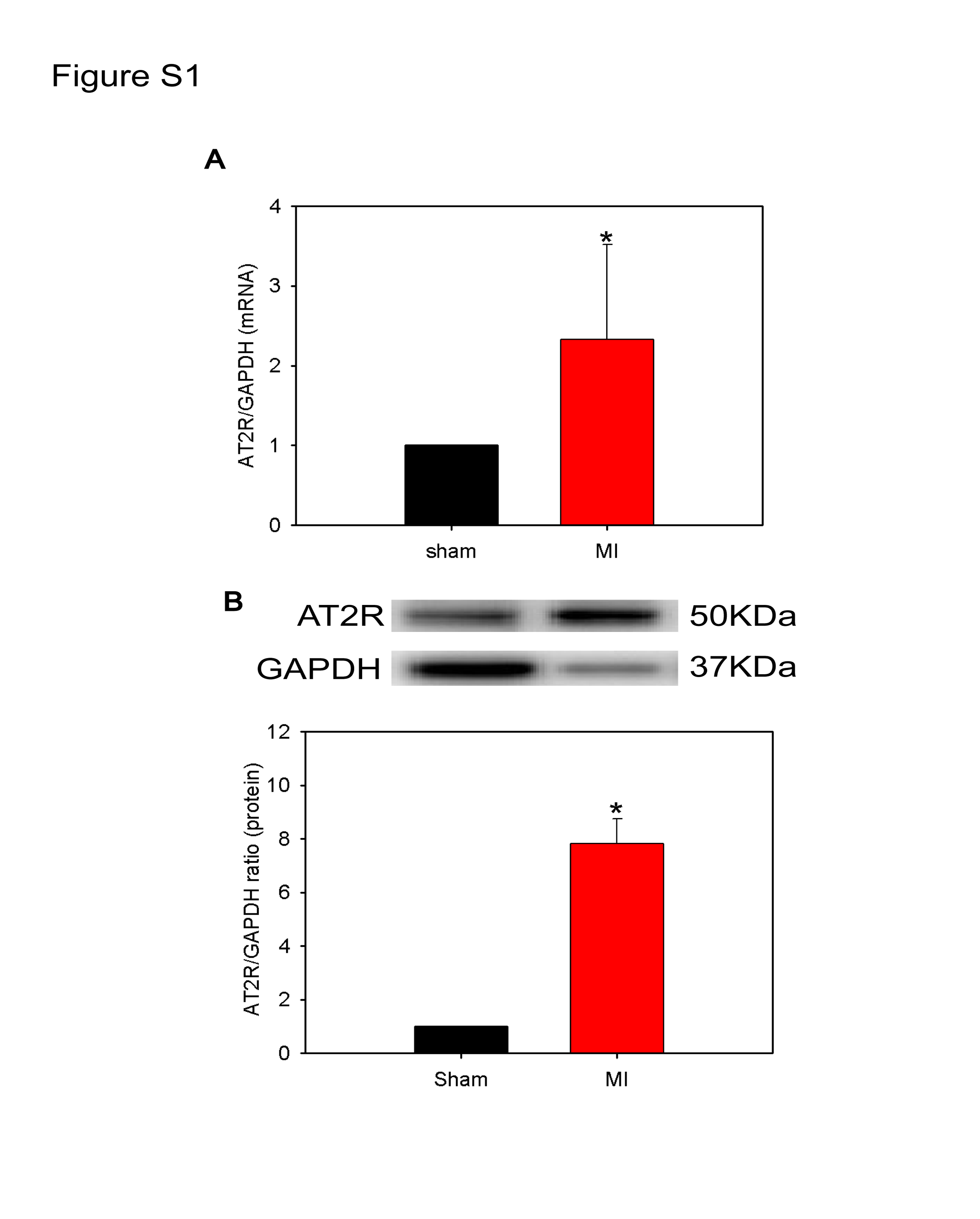

Supplement: Figure S1 — Elevated AT2R Expression in BMMNCs 7days After MI. (A) Real-time PCR for AT2R expression in mononuclear cells that were isolated from rat bone marrow on day 7 after MI. Sham n=6; MI n=6; *P < 0.05 versus sham group. (B) Western blot analysis of AT2R expression level of BMMNCs on day 7 after MI. Sham n=3; MI n=3; *P < 0.05 versus sham group. (TIF) [file pone.0082997.s001.tif]

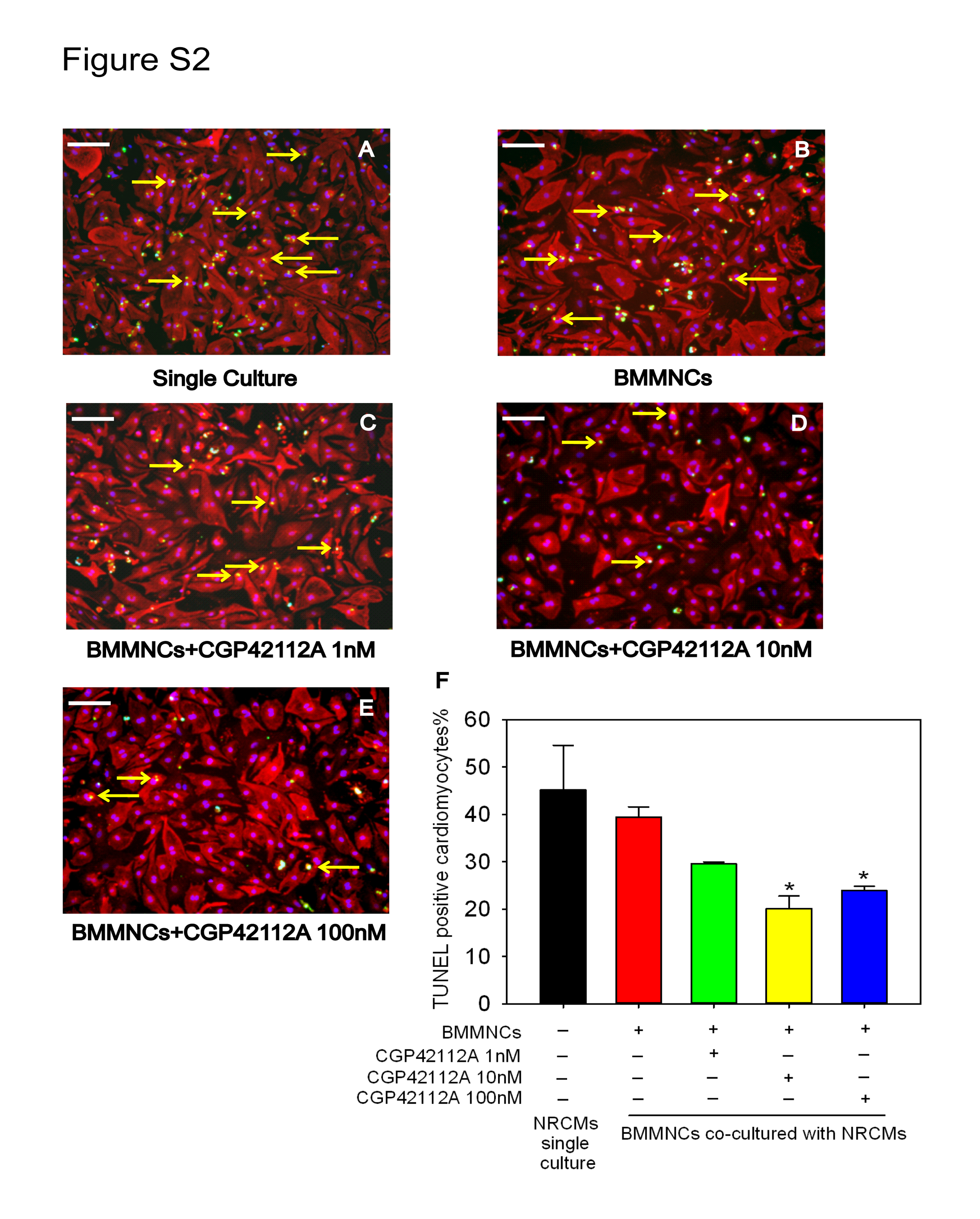

Supplement: Figure S2 — Determination of the Optimal Dosage of CGP42112A for BMMNCs Preconditioning. (A to E) BMMNCs were initially pre-incubated with DMEM, 1nM CGP42112A, 10nM CGP42112A, 100nM CGP42112A for 2 hours at 37 °C, respectively. Then preconditioned BMMNCs were co-cultured with NRCMs under hypoxia in serum free medium for 48 hours. Apoptotic NRCMs were detected using TUNEL assay. Bar=100μm; Green represents TUNEL positive cells; Red represents Troponin T (TnT); Blue represents nuclei; and yellow arrowhead represents apoptotic NRCMs. (F) Quantification of apoptotic NRCMs. n=3 for each group; *P < 0.05 versus BMMNCs group. (TIF) [file pone.0082997.s002.tif]

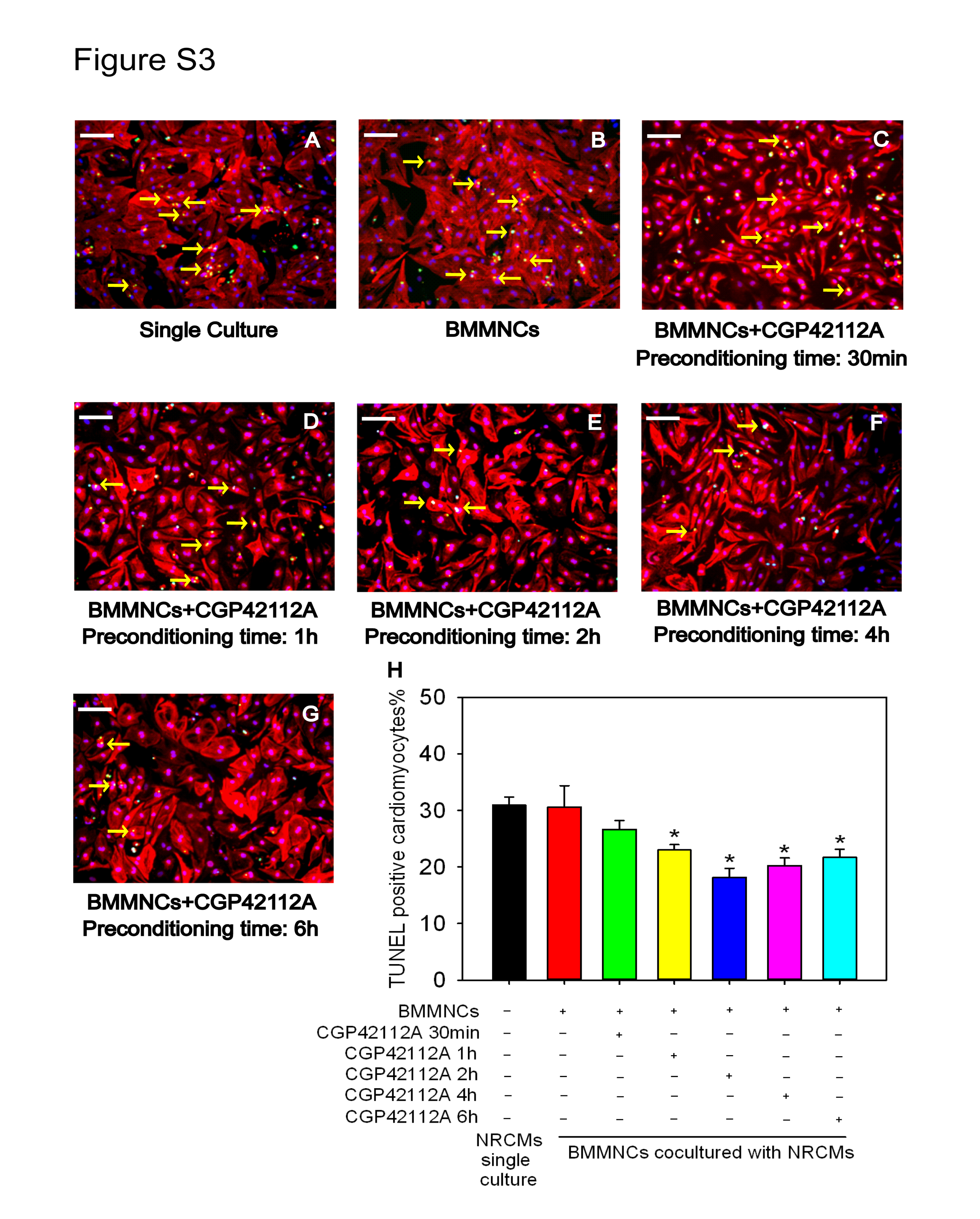

Supplement: Figure S3 — Determination of the Optimal Time Course of CGP42112A for BMMNCs Preconditioning. (A to G) BMMNCs were initially pre-incubated with 10 nM CGP42112A at different time points (30 min, 1h, 2h, 4h, 6h), respectively. Then preconditioned BMMNCs were co-cultured with NRCMs under hypoxia in serum free medium for 48 hours. Apoptotic NRCMs were detected using TUNEL assay. Bar=100μm; Green represents TUNEL positive cells; Red represents Troponin T (TnT); Blue represents nuclei; and yellow arrowhead represents apoptotic NRCMs. (F) Quantification of apoptotic NRCMs. n=3 for each group; *P < 0.05 versus BMMNCs group. (TIF) [file pone.0082997.s003.tif]

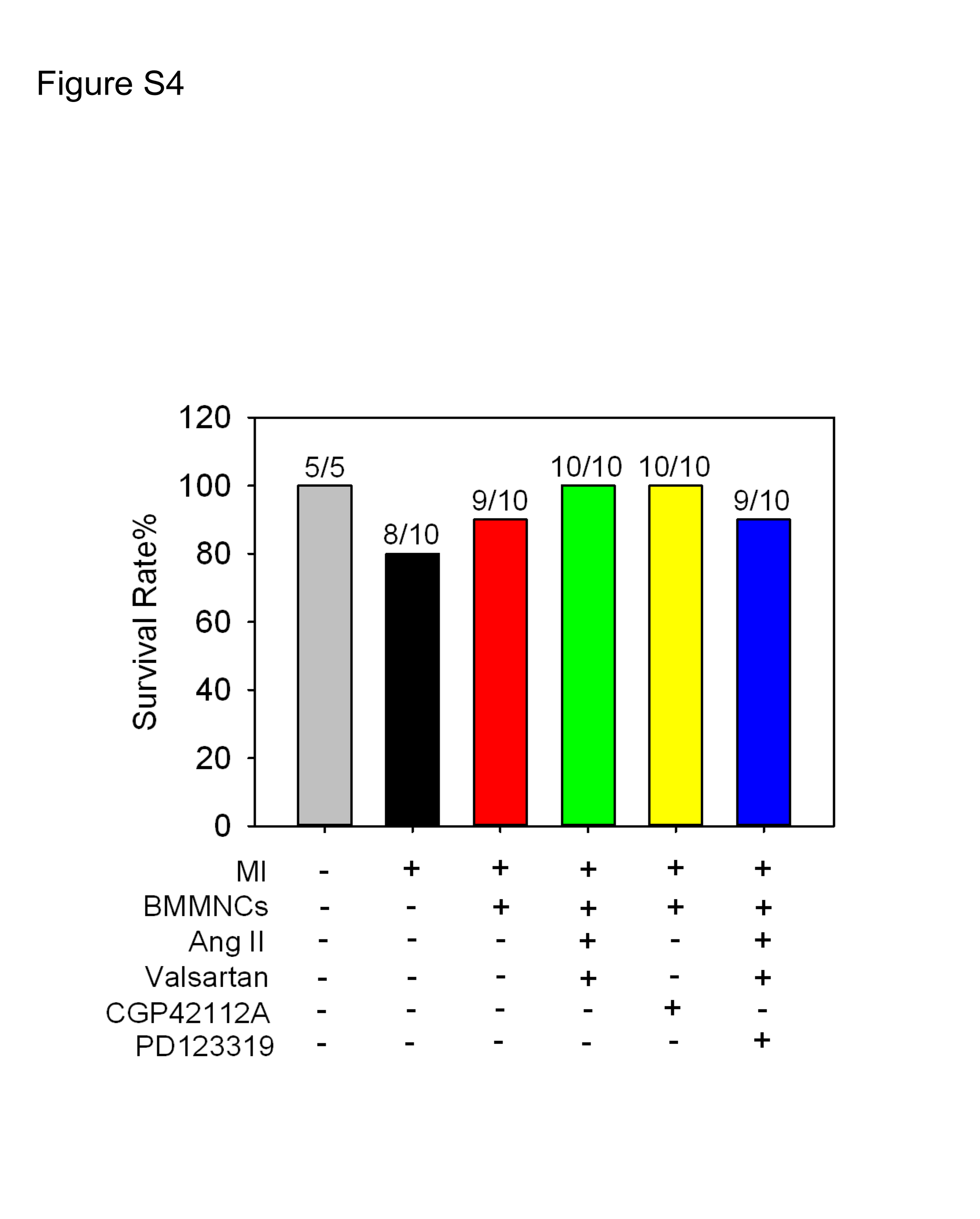

Supplement: Figure S4 — The Animal Survival Ratio Among Each Group During Whole Observation Period. (TIF) [file pone.0082997.s004.tif]

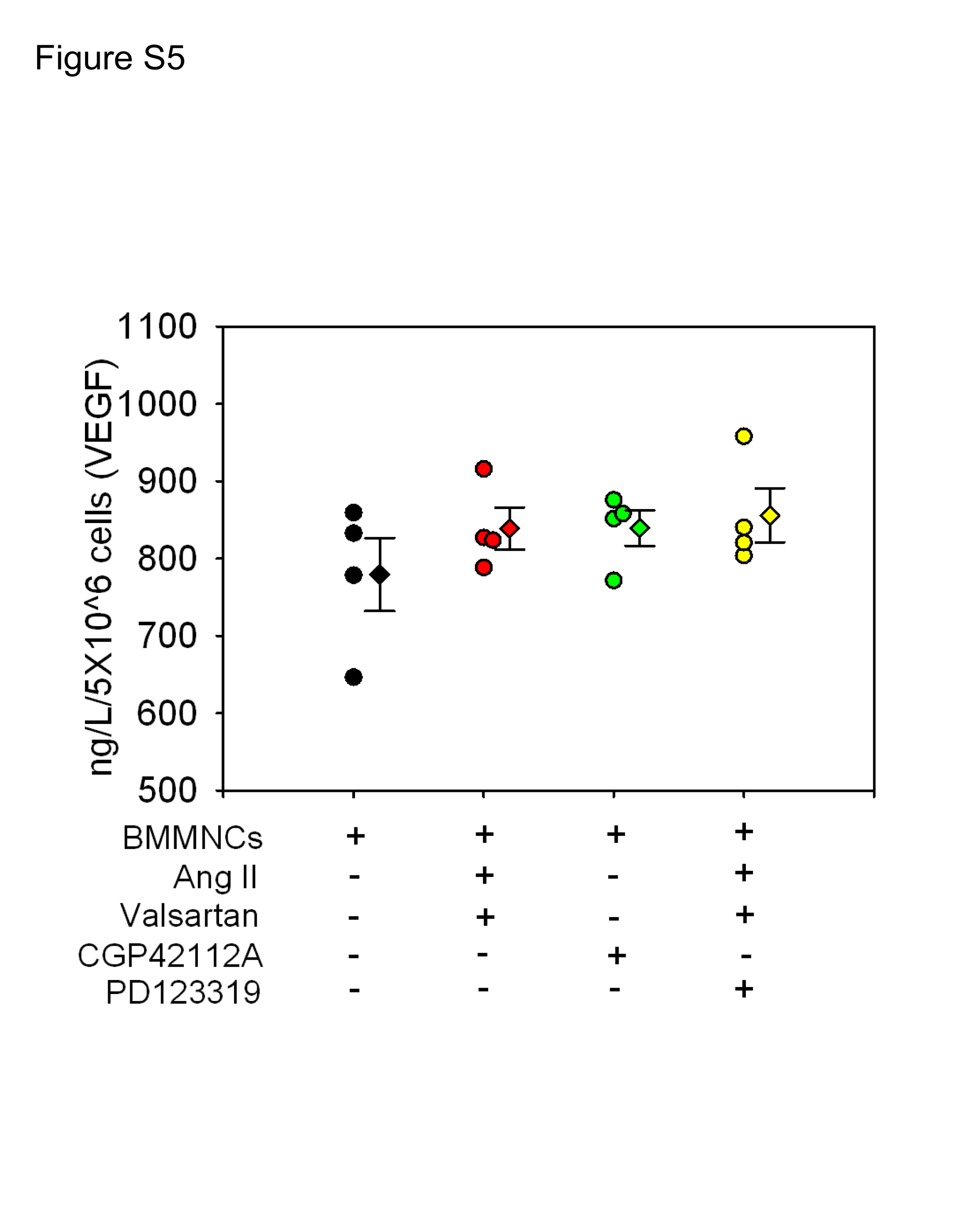

Supplement: Figure S5 — Effect of AT2R Activation on VEGF Secretion by BMMNCs in vitro. BMMNCs were incubated with DMEM, CGP42112A, AngII+Valsartan, or AngII+Valsartan+PD123319 for 2 hours at 37 °C, respectively. Then preconditioned BMMNCs were cultured for 24 hours and medium was collected. Concentration of VEGF in supernatant was measured by ELISA. n=4 for each group. (TIF) [file pone.0082997.s005.tif]

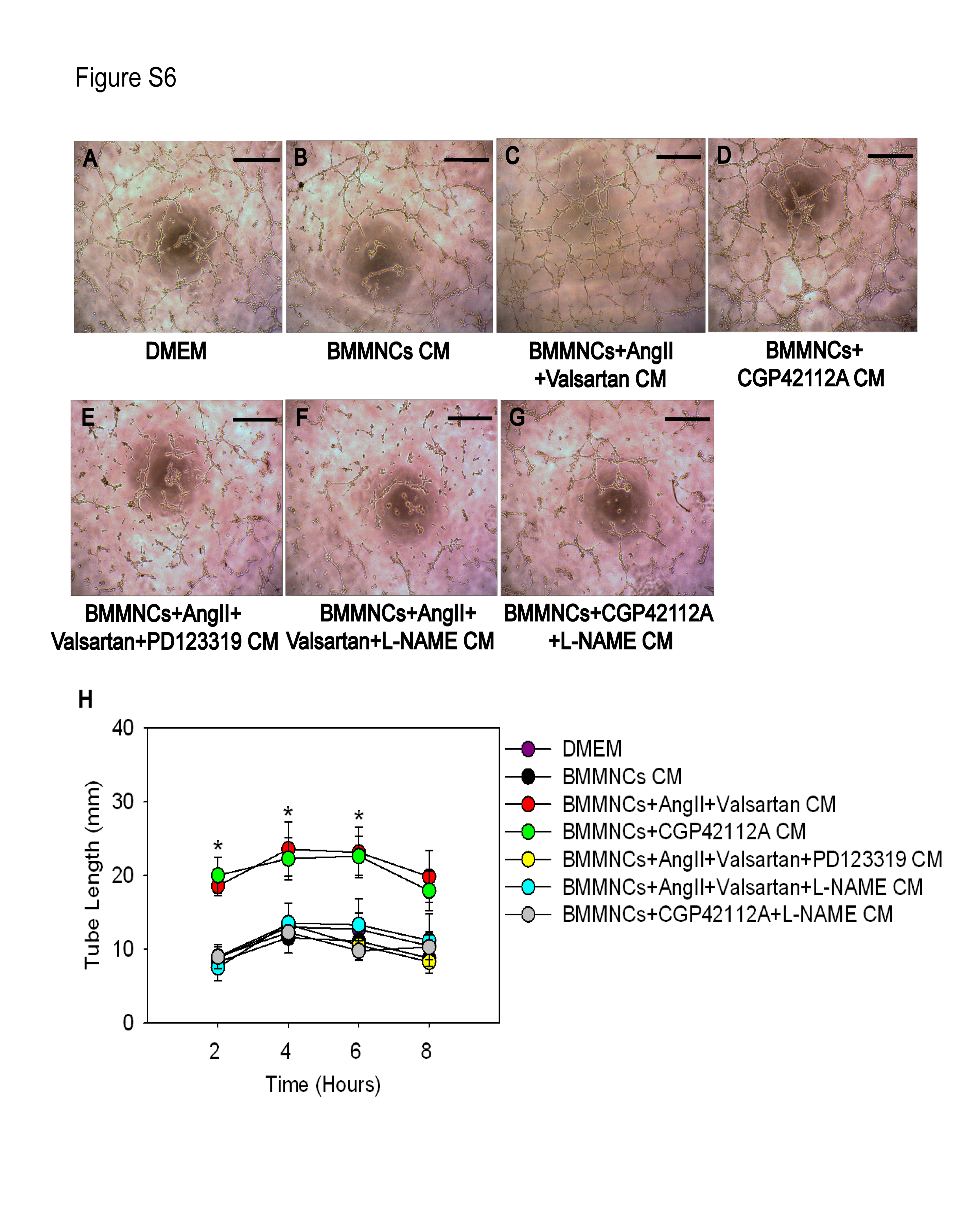

Supplement: Figure S6 — Conditioned Medium (CM) Derived from AT2R Stimulated BMMNCs Enhanced Tube Formation in vitro. (A to G) Representative figures showed tube formation of HUVECs at time point of 6 hours. Bar=500μm. (H) Quantitative analysis of tube length at each time point. n=3 for each group. *P < 0.05 denotes (BMMNCs+AngII+Valsartan CM group or BMMNCs+CGP42112A CM group) versus all other groups by Two Way ANOVA. (TIF) [file pone.0082997.s006.tif]

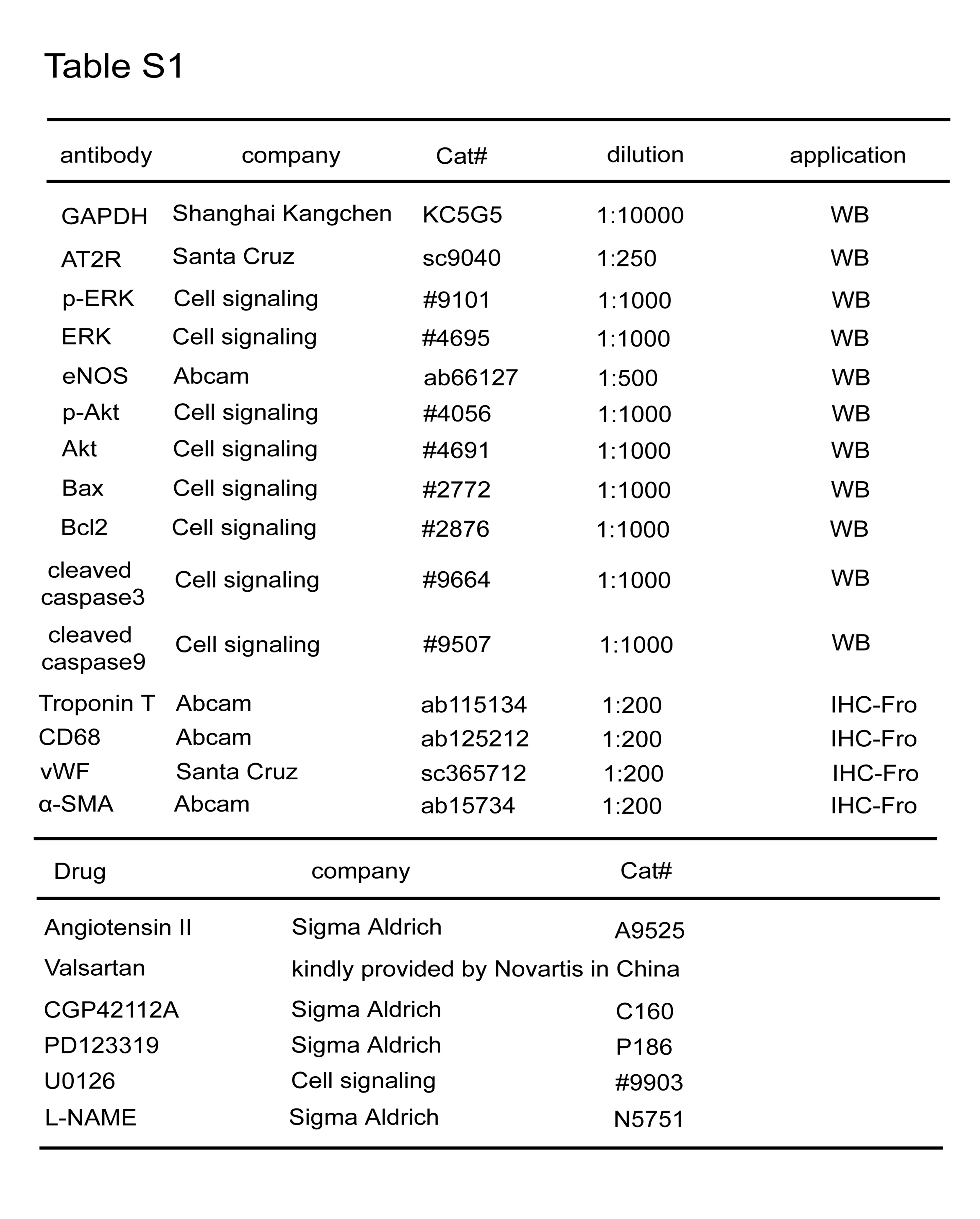

Supplement: Table S1 — Materials Used in Study. (TIF) [file pone.0082997.s007.tif]

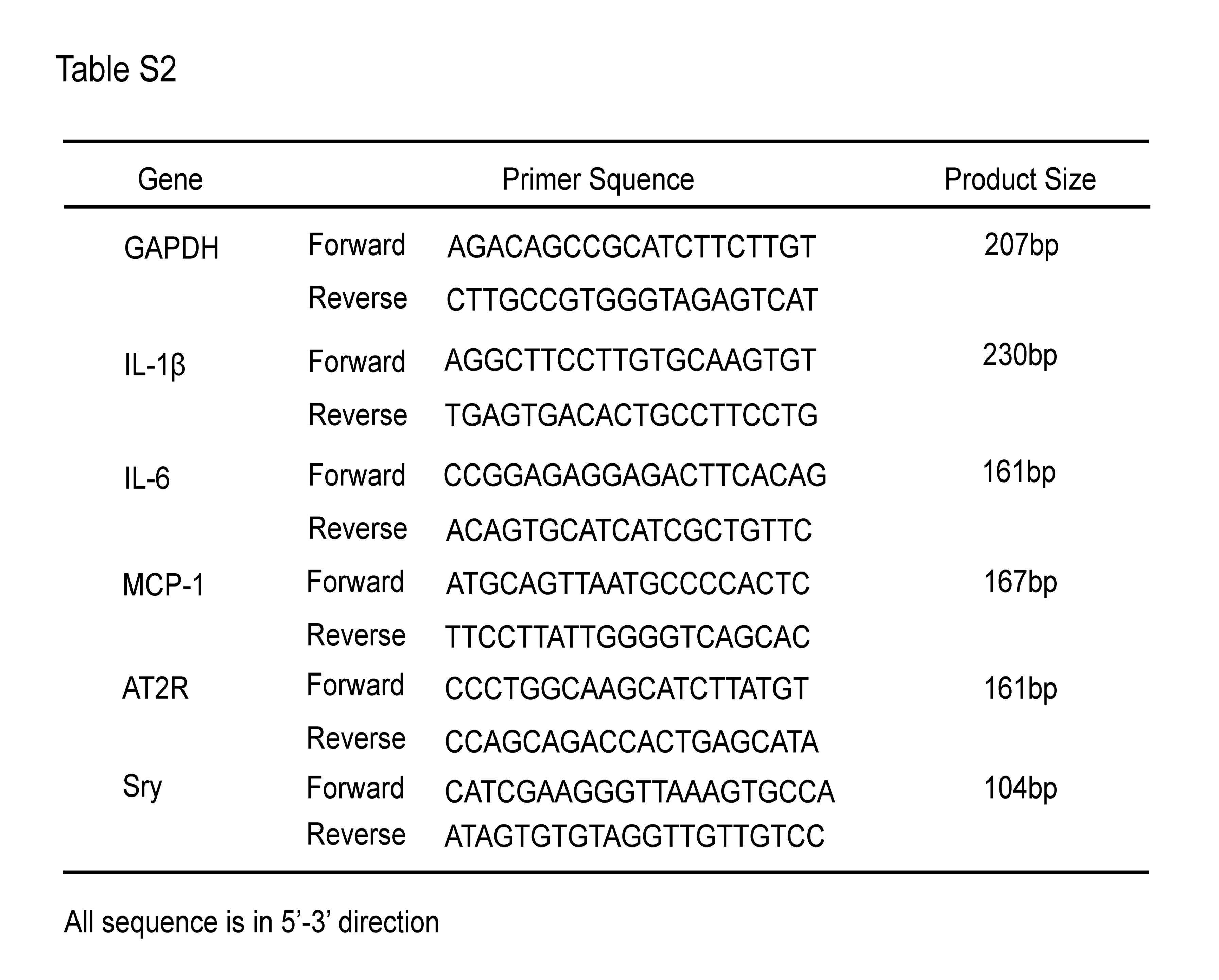

Supplement: Table S2 — Primers Used in Study. (TIF) [file pone.0082997.s008.tif]
